# A New Cyclic Hexapeptide and a New Isocoumarin Derivative from the Marine Sponge-Associated Fungus *Aspergillus similanensis* KUFA 0013

**DOI:** 10.3390/md13031432

**Published:** 2015-03-17

**Authors:** Chadaporn Prompanya, Carla Fernandes, Sara Cravo, Madalena M. M. Pinto, Tida Dethoup, Artur M. S. Silva, Anake Kijjoa

**Affiliations:** 1 ICBAS-Instituto de Ciências Biomédicas Abel Salazar, Universidade do Porto, Rua de Jorge Viterbo Ferreira, 228, 4050-313 Porto, Portugal; E-Mail: chadaporn@buu.ac.th; 2 Interdisciplinary Centre of Marine and Environmental Research (CIIMAR), Rua dos Bragas 289, 4050-123 Porto, Portugal; 3 Centro de Química Medicinal da Universidade do Porto (CEQUIMED-UP, Laboratório de Química Orgânica, Departamento de Ciências Químicas, Faculdade de Farmácia, Rua de Jorge Viterbo Ferreira, 228, 4050-313 Porto, Portugal; E-Mails: cfernandes@ff.up.pt (C.F.); scravo@ff.up.pt (S.C.); madalena@ff.up.pt (M.M.M.P.); 4 Department of Plant Pathology, Faculty of Agriculture, Kasetsart University, Bangkok 10240, Thailand; E-Mail: agrtdd@ku.ac.th; 5 Departamento de Química & QOPNA, Universidade de Aveiro, 3810-193 Aveiro, Portugal; E-Mail: artur.silva@ua.pt

**Keywords:** *Aspergillus similanensis*, cyclic hexapeptide, similanamide, isocoumarin, similanpyrone C, pyripyropene T

## Abstract

A new isocoumarin derivative, similanpyrone C (**1**), a new cyclohexapeptide, similanamide (**2**), and a new pyripyropene derivative, named pyripyropene T (**3**) were isolated from the ethyl acetate extract of the culture of the marine sponge-associated fungus *Aspergillus similanensis* KUFA 0013. The structures of the compounds were established based on 1D and 2D NMR spectral analysis, and in the case of compound **2** the stereochemistry of its amino acid constituents was determined by chiral HPLC analysis of the hydrolysate by co-injection with the d and l amino acids standards. Compounds **2** and **3** were evaluated for their *in vitro* growth inhibitory activity against MCF-7 (breast adenocarcinoma), NCI-H460 (non-small cell lung cancer) and A373 (melanoma) cell lines, as well as antibacterial activity against reference strains and the environmental multidrug-resistant isolates (MRS and VRE). Only compound **2** exhibited weak activity against the three cancer cell lines, and neither of them showed antibacterial activity.

## 1. Introduction

In recent years, there has been an increasing interest in marine-derived fungi as a target source of bioactive marine natural products because many consider them among the world’s greatest untapped resources for new biodiversity as well as chemodiversity [1,2,3]. Moreover, through established culture methods, the compounds can be produced in quantity needed for medicinal chemistry development, clinical trials and even marketing. Among the marine fungal strains investigated, the fungi of the genus *Aspergillus* are the most prolific source of bioactive secondary metabolites, including sterols [4], cerebrosides [5], sesquiterpenoids [6,7], sesterterpenoids [8,9], diterpenoids [10], meroterpenoids [11], anthraquinone derivatives [12,13], nucleoside derivatives [14], indole alkaloids [15,16,17], prenylated indole alkaloids [18,19,20,21], quinazolinone alkaloids [22,23], pyrrolidine alkaloids [8], and cyclic peptides [24,25,26,27,28].

**Figure 1:**
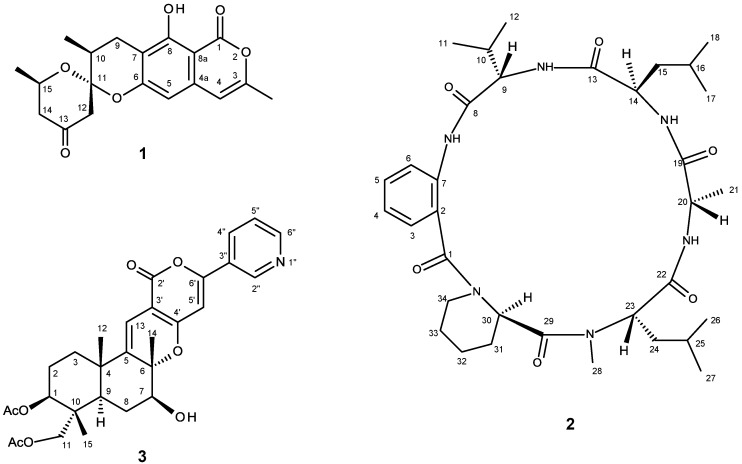
Secondary metabolites from *Aspergillus similanensis* KUFA0013.

In our ongoing search for new natural products with antibacterial and anticancer activities produced by the marine-derived fungi of the genera *Neosartorya* and *Aspergillus*, we have recently reported the isolation of new isocoumarins similanpyrones A and B, a new chevalone (chevalone E), and a new natural product pyripyrone S; besides the previously reported chevalone B and C, a meroterpenoid S14-95, and pyripyropene E, from the crude ethyl acetate extract of the undescribed marine sponge-associated fungus *Aspergillus similanensis* KUFA 0013 [29]. Reexamination of the fractions remaining from the previous study of this fungus led to the isolation of a new 8-hydroxy-3-methylisocoumarin derivative, which we have named similanpyrone C (**1**), a new cyclic hexapeptide, similanamide (**2**), and a new pyripyropene analog, pyripyropene T (**3**) (Figure 1). Hydrolysis of compound **2**, followed by HPLC analysis of its hydrolysate using a chiral column, led to the elucidation of its amino acid constituents. Compounds **2** and **3** were evaluated for their antibacterial activity as well their cytotoxicity against three human cancer cell lines.

## 2. Results and Discussion

Compound **1** was isolated as pale yellow viscous mass, and its molecular formula C_19_H_20_O_6_ was established on the basis of the (+)-HRESIMS *m/z* 345.1342 [M + H]^+^, indicating ten degrees of unsaturation. The IR spectrum showed absorption bands for hydroxyl (3443 cm^−1^), ketone carbonyl (1730 cm^−1^), conjugated lactone carbonyl (1683 cm^−1^), olefin (1647 cm^−1^) and aromatic (1625, 1572 cm^−1^) groups. The ^13^C NMR (Supplementary Information, Figure S3), DEPTs and HSQC spectra (Table 1, Supplementary Information, Figure S4) revealed the presence of one ketone carbonyl (δ_C_ 205.3), one conjugated ester carbonyl (δ_C_ 166.8), six quaternary sp^2^ (δ_C_ 160.0, 158.3, 153.4, 136.7, 110.0 and 99.6), two methine sp^2^ (δ_C_ 104.4 and 103.0), one ketal (δ_C_ 102.9), two sp^3^ methine (δ_C_ 67.2 and 33.9), two sp^3^ methylene (δ_C_ 48.3 and 47.2), and three methyl (δ_C_ 21.6, 19.4 and 15.9) carbons. The ^1^H NMR spectrum (Supplementary Information, Figure S1) revealed, besides a singlet of the hydrogen bonded hydroxyl proton at δ_H_ 11.35, one doublet at δ_H_ 6.13 (*J* = 0.9 Hz) and one singlet at δ_H_ 6.26, two multiplets at δ_H_ 4.15 and δ_H_ 1.98, two double double doublets at δ_H_ 2.48 (*J* = 14.7, 2.9, 1.9 Hz) and δ_H_ 2.28 (*J* = 14.7, 11.3, 0.7 Hz), two double doublets at δ_H_ 2.81 (*J* = 16.8, 5.6 Hz) and δ_H_ 2.55 (14.0, 1.9), two broad doublets at δ_H_ 2.81 (*J* = 14.0 Hz) and δ_H_ 2.57 (*J* = 16.8 Hz), one methyl singlet at δ_H_ 2.24, and two methyl doublets at δ_H_ 1.23 (*J* = 6.2 Hz) and δ_H_ 1.21 (*J* = 6.2 Hz).

**Table 1:** ^1^H and ^13^C NMR (CDCl_3_, 500.13 MHz and 125.8 MHz) and HMBC assignment for **1**.

Position	δ_C_, Type	δ_H_, (*J* in Hz)	COSY	HMBC	NOESY
1	166.8, CO	-			
3	153.4, C	-			
4	104.4, CH	6.13, d (0.9)	CH_3_-3	C-3, 5, 8a	CH_3_-3
4a	136.7, C	-			
5	103.0, CH	6.26, s		C-4, 6, 7, 8a	
6	158.3, C	-			
7	110.0, C	-			
8	160.0, C	-			
8a	99.6, C	-			
9α	23.6, CH_2_	2.81, dd (16.8, 5.6)	H-9β, H-10	C-6, 7, 10, 11	H-9β, H-10, CH_3_-10
9β		2.57, brd (16.8)	H-9α, H-10	C-7, 10, 11	H-9α, H-10, CH_3_-10
10	33.9, CH	1.98, m	H-9α, H-9β, CH_3_-10		H-9α,H-9β, CH_3_-10
11	102.9, C	-			
12α	47.2, CH_2_	2.55, dd (14.0, 1.9)	H-12β	C-10, 11, 13	H-12β
12β		2.81, brd (14.0)	H-12α	C-10, 11, 13	H-12α
13	205.3, CO	-			
14α	48.3, CH_2_	2.28, ddd (14.7, 11.3, 0.7)	H-14β, H-15	C-13, 15	H-14β, CH_3_-15
14β		2.48, ddd (14.7, 2.9, 1.9)	H-14α, H-15	C-13	H-14β, CH_3_-15
15	67.2, CH	4.15, m	H-14α, H-14β, CH_3_-15		H-14β, CH_3_-15
CH_3_-3	19.4, CH_3_	2.24, s	H-4	C-3, 4	H-4
CH_3_-10	15.9, CH_3_	1.23, d (6.2)	H-10	C-9, 10, 11	H-9β, H-10, H-12α, 2β
CH_3_-15	21.6, CH_3_	1.21, d (6.2)	H-15	C-14, 15	H-14α, H-14β, H-15
OH-8	-	11.35, s		C-7, 8, 8a	

**Figure 2:**
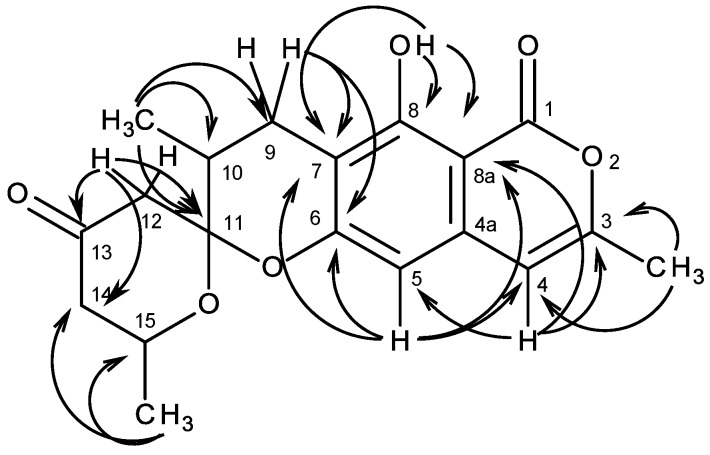
Key HMBC correlations of compound **1**.

Analysis of the ^1^H, ^13^C NMR, HSQC and HMBC spectra (Table 1) revealed the presence of a 6,7-disubstituted 3-methyl-1*H*-isochromen-1-one nucleus, similar to that of similanpyrone B [29]. Thus, another portion of the molecule consisted of one ketone (δ_C_ 205.3), one ketal (δ_C_ 102.9), one methine (δ_H_ 1.98, m; δ_C_ 33.9), one oxymethine (δ_H_ 4.15, m; δ_C_ 67.2), three methylene (δ_H_ 2.81, dd, *J* = 16.8, 5.6 Hz and δ_H_ 2.57, brd, *J* = 16.8 Hz, δ_C_ 23.6; δ_H_ 2.81, brd, *J* = 14.0 Hz and 2.55, dd, *J* = 14.0, 1.9 Hz; δ_C_ 47.2; δ_H_ 2.48, ddd, *J* = 14.7, 2.9, 1.9 Hz and 2.28, ddd, *J* = 14.7, 11.3, 0.7 Hz, δ_C_ 48.3), two methyl (δ_H_ 1.21, d, *J* = 6.2 Hz; δ_C_ 21.6 and δ_H_ 1.23, d, *J* = 6.2 Hz, δ_C_ 15.9) groups. That this portion was 2,10-dimethyl-1,7-dioxaspiro[5.5]undec-8-en-4-one was evidenced by the COSY correlations (Table 1, Supplementary Information, Figure S2) of H_2_-9 (δ_H_ 2.81, dd, *J =* 16.8, 5.6 Hz and 2.57, brd, *J* = 16.8 Hz) to H-10 (δ_H_ 1.98, m), of H-10 to CH_3_-10 (δ_H_ 1.23, d, *J* = 6.2 Hz), of H_2_-14 (δ_H_ 2.48, ddd, *J* = 14.7, 2.9, 1.9 Hz and 2.28, ddd, *J* = 14.7, 11.3, 0.7 Hz) to H-15 (δ_H_ 4.15, m), and of H-15 to CH_3_-15 (δ_H_ 1.21, d, *J* = 6.2 Hz), as well as by the HMBC cross peaks (Table 1, Supplementary Information, Figure S5) of H_2_-9 to C-10 and C-11 (δ_C_ 102.9), of H_2_-12 (δ_H_ 2.81, brd, *J* = 14.0 Hz and 2.55, dd, *J* = 14.0, 1.9 Hz) to C-10, 11, 13 (δ_C_ 205.3), and of H-14 to C-13 and 15, respectively (Figure 2). That the 1,7-dioxaspiro[5.5]undec-8-en-4-one ring system was fused with the 3-methyl-1*H*-isochromen-1-one nucleus, through C-8 and C-9 of the methyldihydropyran ring of the former and C-6 and C-7 of the latter, was supported by the HMBC correlations of H_2_-9 to C-6, 7 (Table 1, Supplementary Information, Figure S5). Literature search revealed that **1** is a new compound, and in compliance with our previous work, we have therefore named it similanpyrone C. Since **1** was isolated as viscous mass, it was not possible to obtain suitable crystals for the X-ray analysis. Consequently, the absolute configuration of C-10, C-11 and C-15 is still undetermined.

In an attempt to determine the stereochemistry of C-10 and C-15, we have sorted out the coupling constants of both H_2_-9 and H_2_-14. The fact that H_2_-9 appeared as a double doublet at δ_H_ 2.81 with a germinal coupling of 16.8 Hz, and a small coupling at δ_H_ 5.6 Hz, typical of axial-equatorial coupling, and a broad doublet at δ_H_ 2.57 with a germinal coupling constant of 16.8 Hz, we concluded that CH_3_-10 was in the β-axial position of the half-chair conformation of the tetrahydropyran ring. This was corroborated by the correlations of H-10, which was in α equatorial position, to both H-9β (δ_H_ 2.57, brd, *J* = 16.8 Hz) and H-9α (δ_H_ 2.81, dd, *J* = 16.8, 5.6 Hz) in the NOESY spectrum (Table 1, Supplementary Information, Figure S6). On the contrary, one of H-14 appeared as a double double doublet at δ_H_ 2.28, with a germinal coupling of 14.7 Hz and a diaxial coupling of 11.3 Hz, as well as a small coupling (long range) of 0.7 Hz, while another appeared also as a double double doublet at δ_H_ 2.48, with a germinal coupling of 14.7 Hz and an axial-equatorial coupling of 2.9 Hz, as well as a small coupling (long range) of 1.9 Hz. These patterns of couplings revealed that CH_3_-15 was in α equatorial position of the chair conformation of the tetrahydro-4*H*-pyran-4-one ring. This analysis was corroborated by the NOESY spectrum (Table 1, Supplementary Information, Figure S6), which exhibited a correlation only between H-15 and H-14β (δ_H_ 2.48, ddd, *J* = 14.7, 2.9, 1.9 Hz) and not between H-15 and H-14α (δ_H_ 2.28, ddd, *J* = 14.7, 11.3, 0.7 Hz). This assignment was also supported by the higher chemical shift value of H-14β than that of H-14α since the former is under the anisotropic deshielding of the C-13 carbonyl group. Based on the same reasoning, we assigned the broad doublet at δ_H_ 2.81 *(J* = 14.0 Hz) as H-12β, and the double doublet at δ_H_ 2.55 *(J* = 14.0, 1.9 Hz) as H-12α. Consequently, the relative configuration of C-10 and C-15 was tentatively assigned as 10S* and 15R*. The relative configuration of C-11 was tentatively assigned as 11S* based on the fact that the NOESY spectrum (Table 1, Supplementary Information, Figure S6) did not exhibit any correlation between H-10 and H-15. According to the molecular model, when the configuration of C-11 is S*, the substituents on C-11 are arranged in a way that H-10 and H-15 are pointing toward the opposite directions. On the contrary, the R* configuration of C-11 would have H-10 and H-15 close enough to give a strong NOESY correlation.

**Scheme 1:**
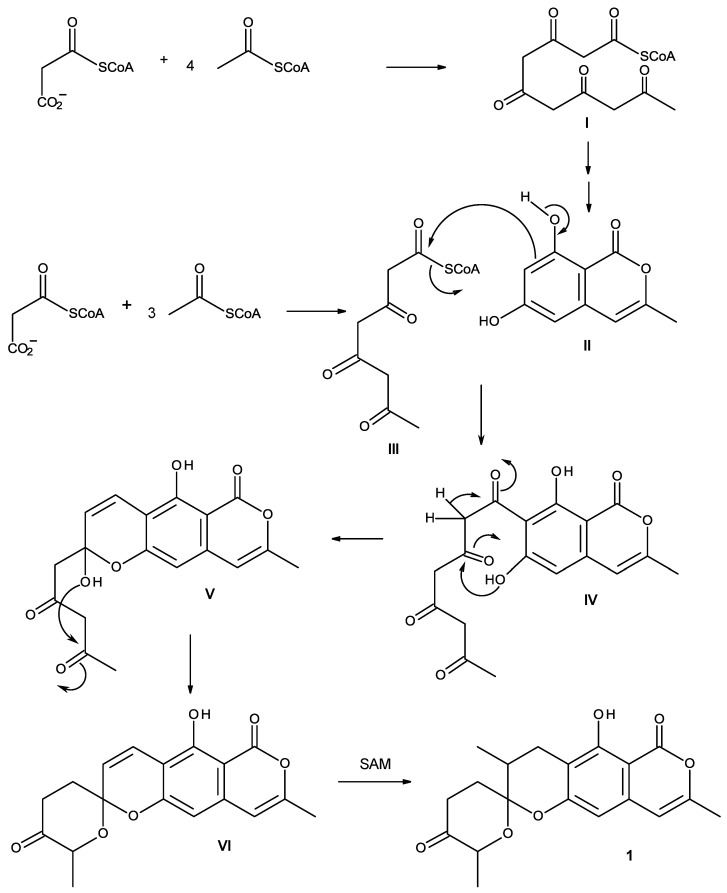
Proposed biogenesis of similanpyrone C (**1**).

Similanpyrone C (**1**) can be assumed to be derived from the acetate-malonate pathway (Scheme 1). Cyclization and enolization of the pentaketide (**I**) leads to the formation of 6,8-dihydroxy-3-methylisocoumarin (**II**), which, after Claisen condensation with the tetraketide (**III**), gives rise to **IV**. Enolization of the side chain, together with a formation of the hemiketal by the phenolic hydroxyl group on C-6 of the isocoumarin nucleus and the ketone carbonyl of the side chain, leads to the formation of a hemiketal **V**. Formation of the ketal and methylation by SAM in the side chain finally gives rise to similanpyrone C (**1**).

**Figure 3:**
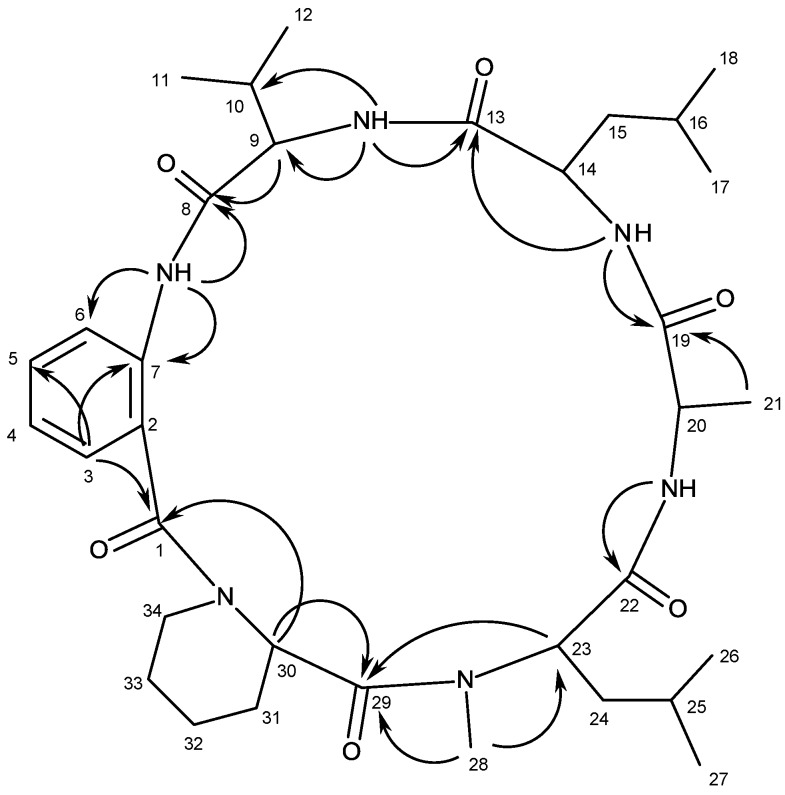
Key HMBC correlations of compound **2**.

Compound **2** was isolated as pale yellow viscous mass, and its molecular formula C_34_H_52_N_6_O6 was established on the basis of the (+)-HRESIMS *m/z* 641.4053 [M + H]^+^, indicating twelve degrees of unsaturation. The IR spectrum showed absorption bands for amine (3335 cm^−1^), carbonyl (1682, 1644 cm^−1^) and aromatic (1594, 1519 cm^−1^). The ^13^C NMR (Supplementary Information, Figure S8), DEPTs and HSQC spectra (Table 2, Supplementary Information, Figure 10S) revealed the presence of six amide carbonyls (δ_C_ 174.3, 174.2, 170.7, 170.2, 169.3, 168.9), two quaternary sp^2^ (δ_C_ 137.0, 122.7), four methine sp^2^ (δ_C_ 131.7, 127.1, 123.9, 123.4), eight methine sp^3^ (δ_C_ 65.1, 61.4, 59.3, 50.9, 47.9, 29.9, 25.5, 24.4), six methylene sp^3^ (δ_C_ 52.5, 37.8, 36.2, 28.1, 27.4, 24.5) and eight methyl (δ_C_ 37.9, 23.3, 23.2, 22.1, 21.7, 19.8, 18.4, 16.2) carbons. The ^1^H NMR spectrum (Table 2, Supplementary Information, Figure S7) revealed, besides four NH signals at δ_H_ 7.43, d (*J* = 7.4 Hz), 7.64, d (*J* = 9.8 Hz), 8.02, d (*J* = 7.9 Hz) and 9.41, brs, the signals of the aromatic protons of the 1,2-disubstituted benzene ring at δ_H_ 7.20, dd (*J* = 7.7, 1.5 Hz), 7.13, ddd (*J* = 7.9, 7.9, 1.0 Hz), 7.47, ddd, (*J* = 7.9, 7.9, 1.6 Hz) and 8.29, d (*J* = 8.3 Hz). That the 1,2-disubstituted benzene ring belonged to the anthranilic acid residue was corroborated by the HMBC correlations of the NH signal at δ_H_ 9.41, brs to the carbon signal at δ_C_ 123.9 (C-6), and of the double doublet at δ_H_ 7.20 (*J* = 7.7, 1.5 Hz, H-3) to the carbons at δ_C_ 131.7 (C-5), 137.0 (C-7) and 170.2 (CO-1) (Table 2, Figure 3, Supplementary Information, Figure S11). The anthranilic acid residue was linked to the valine residue, through the amino group of the former and the carboxyl group of the latter, since the HMBC spectrum (Supplementary Information, Figure S11) showed correlations of the NH signal at δ_H_ 9.41, brs to the carbonyl carbon at δ_C_ 170.7 (C-8), of the methine proton at δ_H_ 4.32 dd, *J* = 7.4, 3.3 Hz (H-9) to the methine carbon at δ_C_ 29.9 (C-10), the methyl carbon at δ_C_ 16.2 (C-12) and C-8, and of the NH signal at δ_H_7.43, d (*J* = 7.5 Hz) to C-9 (δ_C_ 59.3) and C-10 (Table 2, Figure 3). The presence of the leucine residue was supported by the coupling system from CH-14 (δ_H_ 4.57, m; δ_C_ 50.9) through CH_3_-17 (δ_H_ 0.97, d, *J* = 6.5 Hz; δ_C_ 23.3) and CH_3_-18 (δ_H_ 0.88, d, *J* = 6.4 Hz; δ_C_ 21.1), and of NH at δ_H_ 8.02 d (*J* = 7.9 Hz) to H-14, as observed in the COSY spectrum (Table 2, Supplementary Information, Figure S9), as well as by the HMBC correlations of the NH signal at δ_H_ 8.02 d (*J* = 7.9 Hz) to C-14 (Table 2, Figure 3). That the valine residue was linked to the leucine residue was supported by the HMBC cross peak between the NH signal of the former (δ_H_7.43, d, *J* = 7.5 Hz) to the signal of the carbonyl carbon (δ_C_174.2, C-13) of the latter. In turn, the leucine residue was linked to the alanine residue, as evidenced by the HMBC cross peaks of the NH signal of the former to the carbonyl carbon signal (δ_C_174.3, C-19) of the latter, and of the proton signal at δ_H_ 4.82, dd, *J* = 9.7, 7.3 Hz (H-20) to C-19 and the methyl carbon at δ_C_ 18.4 (C-21), as well as by the COSY cross peaks of H-20 to CH_3_-21 (δ_H_ 1.29, d, *J* = 7.3 Hz), and of H-20 to NH at δ_H_ 7.64, d (*J* = 7.9 Hz). The presence of the *N*- methyl leucine moiety was evidenced by the coupling system from H-23 (δ_H_ 3.49, dd, *J* = 9.0, 4.7 Hz) through CH_3_-26 (δ_H_ 0.97d, *J* = 6.5; δ_H_ 23.2) and CH_3_-27 (δ_H_ 0.99, d, *J* = 6.5; δ_H_ 22.1), as observed in the COSY spectrum (Table 2, Supplementary Informatscheion, Figure S9), as well as by the HMBC correlations of H-23 to C-22 (δ_C_ 169.3) and CH_3_-28 (δ_C_ 37.9). Finally, the presence of the pipecolic acid residue was supported by the coupling system from CH-30 (δ_H_ 3.71, dd, *J* = 11.3, 2.5; δ_C_ 61.4) through CH_2_-34 (δ_H_ 3.16, dd, *J* = 13.2, 2.3; 4.14, dd, *J* = 14.4, 2.4; δ_C_ 52.5), as observed in the COSY spectrum (Table 2, Supplementary Information, Figure S9). Since both H-30 and CH_3_-28 gave HMBC cross peaks to C-29 (δ_C_ 168.9), the pipecolic acid residue was linked to the *N*-methyl leucine residue through the carboxyl group of the former and a nitrogen atom of the latter. Since **2** presents twelve degrees of unsaturation, the nitrogen atom of the piperidine ring of the pipecolic acid residue was linked to the carbonyl of the anthranilic acid residue. The proposed structure was supported by the NOESY correlations which showed cross peaks of NH at δ_H_ 9.41, brs to H-9, CH_3_-12, of NH at δ_H_ 7.43 (d, *J* = 7.5 Hz) to H-9, CH_3_-11 (δ_H_ 1.06, d, *J* = 6.9 Hz), CH_3_-12, H-14, of NH at δ_H_ 8.02 (d, *J* = 7.9 Hz) to H-14, H-15 (δ_H_ 2.02, m; 1.77, m), CH_3_-17, of NH at δ_H_ 7.64 (d, *J* = 7.9 Hz) to CH_3_-21, H-23, CH_3_-28, of H-3 to H-34β (δ_H_ 4.14, dd, *J* = 14.4, 2.4 Hz), and of H-30 to CH_3_-28 (Table 2, Supplementary Information, Figure S12). Combining this information, it was possible to conclude that **2** was cyclo (anthranilic acid-Val-Leu-Ala-*N*-methyl-Leu-pipecolic acid).

**Table 2:** ^1^H and ^13^C NMR (CDCl_3_, 500.13 MHz and 125.8 MHz) and HMBC assignment for **2**.

	Position	δ_C_, Type	δ_H_, (*J* in Hz)	COSY	HMBC	NOESY
Anthranilic acid	1	170.2, CO	-			
	2	122.7, C	-			
	3	127.1, CH	7.20, dd (7.7, 1.5)	H-4	C-1, 5, 7	H-34
	4	123.4, CH	7.13, ddd (7.9, 7.9, 1.0)	H-3, 5	C-2, 6	
	5	131.7, CH	7.47, ddd (7.9, 7.9, 1.6)	H-4, 6	C-3, 7	
	6	123.9, CH	8.29, d (8.3)	H-5	C-2, 4	H-12
	7	137.0, C	-			
	NH	-	9.41, brs		C-6, 7, 8	NH (Val), H-9, 12
Val	8	170.7, CO	-			
	9	59.3, CH	4.32, dd (7.4, 3.3)	H-10, NH	C-8, 10, 11, 12	H-10, 11
	10	29.9, CH	2.68, m	H-9, 11, 12		H-9, 11, 12
	11	19.8, CH_3_	1.06, d (6.9)	H-10	C-9, 10, 12	
	12	16.2, CH_3_	0.94, d (7.0)	H-10	C-9, 10, 11	
	NH	-	7.43, d (7.5)	H-9	C-9, 10, 13	H-9, 11, 12, 14
Leu	13	174.2, CO	-			
	14	50.9, CH	4.57, m	H-15, NH		H-15, 18
	15	36.2, CH_2_	2.02, m; 1.77, m	H-14, 16		
	16	24.4, CH	1.77, m	H-15, 17, 18		
	17	23.3, CH_3_	0.97, d (6.5)	H-16	C-15, 16, 18	
	18	21.7, CH_3_	0.88, d (6.4)	H-16	C-15, 16, 17	
	NH	-	8.02, d (7.9)	H-14	C-13, 19	NH (Ala), H-14, 15, 17
Ala	19	174.3, CO	-			
	20	47.9, CH	4.82, dd (9.7, 7.3)	H-21, NH	C-19, 21	H-21
	21	18.4, CH_3_	1.29, d (7.3)	H-20	C-19, 20	
	NH	-	7.64, d (7.9)	H-20	C-22	C-21, 23, 28
*N*-Me Leu	22	169.3, CO	-			
	23	65.1, CH	3.49, dd (9.0, 4.7)	H-24	C-22, 24, 28, 29	
	24	37.8, CH_2_	1.95, m; 2.20, m	H-23, 25		
	25	25.5, CH	1.65, m	H-24, 26, 27		
	26	23.2, CH_3_	0.97, d (6.5)	H-25	C-24, 25, 27	
	27	22.1, CH_3_	0.99, d (6.5)	H-25	C-24, 25, 26	
	28	37.9, CH_3_	3.20, s		C-23, 29	23, 30, 32, 34α
Pipecolic acid	29	168.9, CO	-			
	30	61.4, CH	3.71, dd (11.3, 2.5)	H-31	C-1, 29	H-34α
	31	28.1, CH_2_	2.05, m	H-30, 32		
	32	24.5, CH_2_	2.07, m	H-31, 33		
	33	27.4, CH_2_	1.56, m	H-32, 34		
	34α	52.5, CH_2_	3.16, dd (13.2 2.3)	H-33		H-34β
	34β		4.14, dd (14.4, 2.4)			H-34α

The ^1^H and ^13^C NMR data of compound **2** resembled those of PF1171C, a cyclic hexapeptide isolated from extracts of the unidentified ascomycete OK-128 fermented with okara by Kai *et al.* [30], and later by a total synthesis by Masuda *et al.* [31]; however, its value of optical rotation was quite different from that of PF1171C [30,31]. Moreover, PF1171C was reported as white solid (mp 138–140 °C) [31], while compound **2** is pale yellow viscous mass. This observation suggested that compound **2** and PF1171C could be diastereomers.

The stereochemistry of the amino acid residues of compound **2** was determined by chiral HPLC analysis of its acidic hydrolysate, using appropriate d and l amino acids standards, according to the previously described method [32]. The enantioseparations of the standard amino acids were successfully performed with the Chirobiotic T column under reversed-phase elution conditions [33]. Actually, the teicoplanin selector of this column has several characteristic features that make it suitable for amino acid analysis [33,34,35]. The elution order of the enantiomers of all the standard amino acids was confirmed by injecting the solutions of the racemic or enantiomeric mixtures and then each enantiomer separately at a flow rate of 1 mL/min (Supplementary Information, Figure S18). As predicted, the D enantiomer was always more strongly retained than the corresponding L enantiomer on the Chirobiotic T column [33]. Based on mix HPLC analyses of the acidic hydrolysate with standard amino acids (co-injection) (Supplementary Information, Figure S19 and Table S1), compound **2** was elucidated unambiguously as cyclo (anthranilic acid-l-Val-d-Leu-l-Ala-*N*-methyl-l-Leu-d-pipecolic acid). Although the amino acid sequence of compound **2** is the same as that of PF1171C, the stereochemistry of its amino acid constituents is different from that of the amino acids constituent of PF1171C. While PF1171C contains d-Ala, l-Leu, d-Val, and l-pipecolic acid, compound **2** contains l-Ala, d-Leu, l-Val and d-pipecolic acid. Thus compound **2** is a new compound, and we have named it similanamide.

It is interesting to note that Kai *et al.* [30] has firstly assigned the stereochemistry of the amino acid constituents of PF1171C using the reversed phase HPLC analysis of the Marfey derivatives of the amino acids. Since the retention times for the Marfey derivatives of d-Ala (19.4 min) and l-Ala (20.0 min) were too close for resolution, they had wrongly assigned d-Ala for l-Ala. However, in our chiral HPLC analysis using the Chirobiotic T column under reversed-phase elution conditions, not only the retention time of l-Ala (7.16 min) was very different from that of d-Ala (9.36 min), but also the retention times of the d/l pair of other amino acid constituents (Supplementary Information, Table S1).

So far, only few cyclohexapeptides have been reported from marine-derived microorganisms. Wu *et al.* [36] have isolated two cyclohexapeptides, nocardiamides A and B, from the culture broth of the marine-derived actinomycete *Nocardiopsis* sp. CNX037, isolated from sediment. Cai *et al.* [37] have isolated two anti-*Mycobacterium tuberculosis* cyclohexapeptides from a *Streptomyces hygroscopicus* Strain, while Song *et al.* [38] reported isolation of three new cyclopeptides, desotamides B–D, from the deep South China Sea-derived *Streptomyces scopuliridis* SCSIO ZJ46. To the best of our knowledge, compound **2** is the first cyclopeptide containing d-pipecolic acid residue ever isolated from marine fungi.

Compound **3** was isolated as pale yellow viscous mass, and its molecular formula C_29_H_33_NO_8_ was established on the basis of (+)-HRESIMS *m/z* 524.2287 [M + H]^+^, indicating fourteen degrees of unsaturation. The IR spectrum showed absorption bands for hydroxyl (3418 cm^−1^), ester carbonyl (1732 cm^−1^), conjugated ester carbonyl (1667cm^−1^), olefin (1643 cm^−1^), aromatic (1557, 1507 cm^−1^). The ^13^C NMR (Supplementary Information, Figure S15), DEPTs and HSQC spectra (Table 3, Supplementary Information, Figure S16) revealed the presence of two ester carbonyl (δ_C_ 170.1 and 169.8), one conjugated carbonyl (δ_C_ 161.6), five quaternary sp^2^ (δ_C_ 160.2, 156.8, 146.1, 126.9, 100.3), six methine sp^2^ (δ_C_ 151.3, 146.5, 132.8, 123.9, 109.4, 98.7), one oxyquaternary sp^3^ (δ_C_ 85.9), two oxymethine sp^3^ (δ_C_ 75.7 and 72.8), one oxymethylene sp^3^ (δ_C_ 64.4), two quaternary sp^3^ (δ_C_ 40.3, 38.4), one methine sp^3^ (δ_C_ 40.7), three methylene sp^3^ (δ_C_ 35.2, 27.3, 22.9), and five methyl (δ_C_ 23.8, 20.8, 20.5, 20.1 and 12.7) carbons. The general feature of ^13^C and ^1^H spectra of compound **3** closely resembled those of pyripyropene S, previously isolated from the same fungus [29]. Analysis of the ^1^H (Supplementary Information, Figure S13), ^13^C, HSQC and HMBC spectra (Table 3, Supplementary Information, Figure S17) revealed the presence of the ring system comprising of the decahydronaphthalene fused, on C-5 and C-6, with the 2*H*,5*H*-pyrano[4,3-*b*]pyran-5-one, which connected to the pyridine ring through C-6′ of the former and C-3″ of the latter, similar to pyripyropene S [29]. However, there were only two acetoxyl groups (δ_C_ 170.1, CO; δ_C_ 20.5, CH_3_, δ_H_ 2.00, s and δ_C_ 169.8, CO; δ_C_ 20.8, CH_3_; δ_H_ 2.00, s) in compound **3**. That the acetoxyl groups were on C-1 and C-11 was supported by the fact that the chemical shift values of H-1 (δ_H_ 4.64, t, *J* = 8.5 Hz) and H-11 (δ_H_ 3.75, s) were very similar to those of pyripyropene S, whereas the chemical shift of H-7 (δ_H_ 3.85, dd, *J* = 10.6, 4.2 Hz) was nearly 1.4 ppm less than that of pyripyropene S. On the other hand, the ^13^C chemical shift values of C-6 (δ_H_ 85.9) and C-8 (δ_C_ 27.4) of compound **3** were 2.00 and 3.00 ppm, respectively, higher than those of the corresponding carbons in pyripyropene S, while the ^13^C chemical shift value of C-7 (δ_C_ 75.5) of compound **3** was 2.00 ppm lower than that of C-7 of pyripyropene S. Since H-7 appeared as a double doublet with coupling constants of 10.6 and 4.2 Hz, the position of the hydroxyl group on C-7 was β. Thus, compound **3** is 7-deacetylpyripyropene S. In order to prove the stereochemistry of compound **3**, the NOESY experiment was carried out. As the NOESY spectrum (Table 3) clearly exhibited correlations of CH_3_-15 to CH_3_-12, but not to H-1 and H-9; of CH_3_-12 to CH_3_-14 and CH_3_-15, and of CH_3_-14 to CH_3_-12, but not to H-7 (Table 3, Supplementary Information, Figure S18), the stereochemistry of compound **3** is the same as that of pyripyropene S [29], *i.e.*, 1S*, 4R*, 6S*, 7S*, 9R*, 10R*. Since it is a new compound we have named it pyripyropene T.

**Table 3:** ^1^H and ^13^C NMR (DMSO, 300.13 MHz and 75.47 MHz) and HMBC assignment for **3**.

Position	δ_C_, Type	δ_H_, (*J* in Hz)	COSY	HMBC	NOESY
1	72.8, CH	4.64, t (8.5)	H-2		
2	22.9, CH_2_	1.79, m	H-1, 3		
3	35.2, CH_2_	1.98, m	H-2		
4	38.4, C	-			
5	146.1, C	-			
6	85.9, C	-			
7	75.7, CH	3.85, dd (10.6, 4.2)	H-8		
8	27.3, CH_2_	1.70, m	H-7		
9	40.7, CH	1.48, m	H-8		
10	40.3, C	-			
11	64.4, CH_2_	3.75, s		C-1, 9	H_3_-15
12	23.8, CH_3_	1.19, s		C-3, 4, 5	H_3_-14, 15
13	109.4, CH	6.16, s		C-4, 6, 2″, 4″	
14	20.1, CH_3_	1.45, s		C-5, 6, 7	H_3_-12
15	12.7, CH_3_	0.84, s		C-1, 9, 10, 11	H_2_-11, H_3_-12
2′	161.6, C	-			
3′	100.3, C	-			
4′	160.2, C	-			
5′	98.7, CH	7.11, s		C-3′, 4′, 6′, 3″	
6′	156.8, C	-			
2″	146.5, CH	9.0, d (1.7)	H-4″	C-3″, 6″	H-5′
3″	126.9, C	-			
4″	132.8, CH	8.25, dt (8.7, 2.2)	H-2″, 5″		H-5′, 5″
5″	123.9, CH	7.54, dd (7.9, 4.8)	H-4″, 6″	C-3″	H-4″, 6″
6″	151.3, CH	8.68, dd (4.8, 1.5)	H-2″, 5″	C-2″, 4″	H-5″
OAc-1	170.1, CO	-			
	20.5, CH_3_	2.00, s		CO (Ac)	
OAc-11	169.8, CO	-			
	20.8, CH_3_	2.00, s		CO (Ac)	

Since compound **1** was isolated in a very small amount, only compounds **2** and **3** were evaluated for their cytotoxicity and antibacterial activity. Compounds **2** exhibited weak *in vitro* growth inhibitory activity, by Sulforhodamine B (SRB) assay [39], against the MCF-7 (breast adenocarcinoma, GI_50_ = 125 ± 0), NCI-H460 (non-small cell lung cancer, GI_50_ = 117.50 ± 3.55) and A373 (melanoma, GI_50_ = 115 ± 7.07) cell lines. Compounds **2** and **3** were also tested for their antibacterial activity against four reference strains (*Staphylococcus aureus*, *Bacillus subtilis*, *Escherichia coli* and *Pseudomonas aeruginosa*), as well as the environmental multidrug-resistant isolates, according to the previously described method [40], and neither of them showed activity (MIC values higher than 256 µg/mL).

## 3. Experimental Section

### 3.1. General Procedure

Melting points were determined on a Bock monoscope and are uncorrected. Optical rotations were determined on an ADP410 Polarimeter (Bellingham + Stanley Ltd., Tunbridge Wells, Kent, UK). Infrared spectra were recorded in a KBr microplate in a FTIR spectrometer Nicolet iS10 from Thermo Scientific (Waltham, MA, USA.) with Smart OMNI-Transmission accessory (Software 188 OMNIC 8.3). ^1^H and ^13^C-NMR spectra were recorded at ambient temperature on a Bruker AMC instrument (Bruker Biosciences Corporation, Billerica, MA, USA) operating at 500.13 and 125.8 MHz or at 300.13 and 75.4 MHz, respectively. High-resolution mass spectra were measured with a Waters Xevo QToF mass spectrometer (Waters Corporations, Milford, MA, USA) coupled to a Waters Aquity UPLC system. A Merck (Darmstadt, Germany) silica gel GF254 was used for preparative TLC, and a Merck Si gel 60 (0.2–0.5 mm) was used for analytical chromatography.

### 3.2. Extraction and Isolation

Isolation and identification of the fungus as well as fractionation of the crude extract of the culture of *A. similanensis* KUFA0013 have been previously described by us [29]. Frs 185–196 were combined (654 mg) and purified by TLC (Si gel, CHCl_3_:Me_2_CO:HCO_2_H, 97:3:0.1) to give 7.4 mg of **1**. Frs 310–327 were combined (1.19 g), applied on a Sephadex H-20 column (10 g) and eluted MeOH, wherein ten sfrs of 1 mL were collected. Sfrs 1–7 were combined and purified by TLC (Si gel, CHCl_3_:Me_2_CO:HCO_2_H, 19:1:0.01) to give 108 mg of **2**. Frs 336–345 were combined (165 mg) and purified by TLC (Si gel, CHCl_3_:Me_2_CO:HCO_2_H, 17:3:0.01) to give additional 60 mg of **2**. Frs 354–398 were combined (1.15 g), applied on a Sephadex LH-20 column (10 g) and eluted with MeOH, wherein thirty four sfrs of 1 mL were collected. Sfrs 7–15 were combined (150 mg) and purified by TLC (Si gel, CHCl_3_:Me_2_CO:HCO_2_H, 4:1:0.01) to give 67 mg of pyripyropene S [29]. Frs 435–443 were combined (377 mg), applied on a Sephadex LH-20 column (10 g) and eluted with a mixture of 1:1 v/v of CHCl_3_: MeOH, wherein fourteen sfrs of 1 mL were collected. Sfrs 8–11 (62 mg) were combined and purified by TLC (Si gel, CHCl_3_:Me_2_CO:HCO_2_H, 19:1:0.01) to give 35 mg of **3**.

#### 3.2.1. Similanpyrone C (**1**)

Pale yellow viscous mass; [α]_D_^20^ = −80.0 (*c* 0.01, CHCl_3_); λ_max_ (log ε) 239 (4.57), 245(4.59), 332 (2.10) nm; IR (KBr) ν_max_ 3443, 2923, 2852, 1730, 1683, 1647, 1625, 1572, 1508, 1457, 1429, 1352, 1251 cm^−1^; ^1^H and ^13^C NMR (Table 1); HRESIMS *m/z* 345.1342 (M + H)^+^ (calculated for C_19_H_21_O_6_, 345.1338).

#### 3.2.2. Similanamide (**2**)

Pale yellow viscous mass; [α]_D_^20^ = +30.3 (CHCl_3_, *c* 0.03), IR (KBr) ν_max_ 3335, 3054, 2958, 2870, 1682, 1644, 1594, 1519, 1449, 1292 cm^−1^; ^1^H and ^13^C NMR (Table 2); HRESIMS *m/z* 641.4053 (calculated for C_34_H_53_N_6_O_6_, 641.4027).

#### 3.2.3. Pyripyropene T (**3**)

Pale yellow viscous mass; [α]_D_^20^ = +106 (CHCl_3_, *c* 0.03), IR (KBr) ν_max_ 3418, 2949, 1732, 1667, 1643, 1557, 1507, 1480, 1246, 1028 cm^−1^; ^1^H and ^13^C NMR (Table 3); HRESIMS *m/z* 524.2287 (M + H)^+^, calcd for C_29_H_34_NO_8_, 524.2284.

### 3.3. Amino Acids Analysis of Acidic Hydrolysate of Compound **2**

#### 3.3.1. Acid Hydrolysis

The stereochemistry of the amino acids was determined by analysis of the acidic hydrolysate from compound **2**. Compound **2** (5.0 mg) was dissolved in 6 N HCl (5 mL) and heated at 110 °C, in a furnace, for 24 h in a sealed glass tube. After cooling to room temperature, the solution was dried under N_2_ for 24 h, reconstituted in methanol for HPLC-MS (200 µL), filtered through a 4 mm PTFE Syringe Filter F2504-4 of 0.2 µm pore size (Thermo Scientific, Mumbai, India), and then analyzed by HPLC equipped with a chiral column.

#### 3.3.2. Chiral HPLC Analysis

The HPLC system consisted of Shimadzu LC-20AD pump, equipped with a Shimadzu DGV-20A5 degasser, a Rheodyne 7725i injector fitted with a 20 µL loop, and a SPD-M20A DAD detector (Kyoto, Japan). Data acquisition was performed using Shimadzu LCMS Lab Solutions software, version 3.50 SP2. The chiral column used in this study was Chirobiotic T (15 cm × 4.6 mm I.D., particle size 5 µm) manufactured by ASTEC (Whippany, NJ, USA). The mobile phase composition was MeOH:H_2_O:CH_3_CO_2_H (70:30:0.02, v/v/v), all were LC-MS grade solvents obtained from Sigma-Aldrich Co (St. Louis, MO, USA). The flow rate was 0.5 mL/min and the UV detection wavelength was 210 nm. Analyses were performed at room temperature in an isocratic mode.

All standards of racemic amino acids and pure amino acid enantiomers were purchased from Sigma-Aldrich Co (St. Louis, MO, USA). The elution order of the enantiomers of all the standards amino acids was confirmed by injecting the solutions of the racemic or enantiomeric mixtures, and then each enantiomer separately (or only l- amino acid in the case of *N*-methyl leucine) at a flow rate of 1 mL/min or 0.5 mL/min. Working solutions of single enantiomeric amino acids were prepared by dissolution in MeOH at the concentration of 1 mg/mL (10 µL sample injection), while the enantiomeric mixtures were prepared by mixing equal aliquots of each enantiomer (20 µL sample injection). Mix HPLC analyses of the acidic hydrolysate with standard amino acids (co-injection) confirmed the stereochemistry of the amino acids of compound **2**.

## 4. Conclusions

Following our first report of the isolation of new isocoumarin derivatives and merotepenoids from the ethyl acetate crude extract of the culture of the undescribed marine sponge-associated fungus *Aspergillus similanensis* KUFA 0013, we have reexamined its remaining column fractions and have isolated a new isocoumarin derivative containing an unusual 1,7-dioxaspiro-undecenone moiety, together with a new cyclohexapeptide and a new pyripyropene analog. Although several cyclopeptides have been reported from many fungi of the genus *Aspergillus*, this is the first report of isolation of cyclohexapeptide from the marine-derived fungus. The fact that these new cyclohexapeptide and pyripyropene analog did not exhibit relevant antibacterial and the *in vitro* growth inhibitory activities on human cancer cell lines does not mean that they are void of other interesting biological activities. In order to prove this hypothesis, it is necessary to explore their potential in a broader biological or pharmacological assay system.

## Supplementary Files

Supplementary File 1
