# Supplementary material for: A New Cyclic Hexapeptide and a New Isocoumarin Derivative from the Marine Sponge-Associated Fungus Aspergillus similanensis KUFA 0013
Source: Mar Drugs. 2015 Mar 17;13(3):1432–50. doi: 10.3390/md13031432 (PMC4377992; doi:10.3390/md13031432)
Supplement: Supplementary File 1 [file marinedrugs-13-01432-s001.pdf]

Supplementary Information

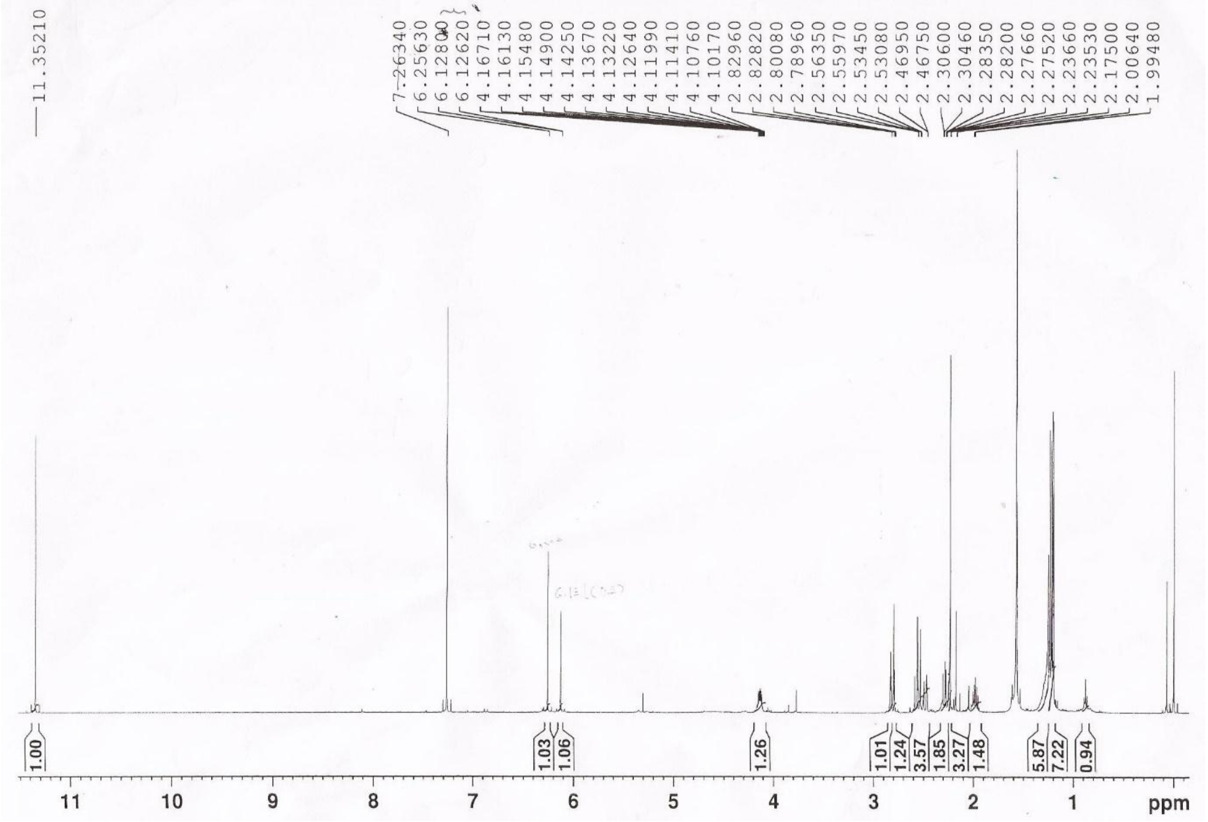

Figure S1. ^{1}H NMR spectrum of compound **1** ($CDCl_3$, 500.13 MHz).

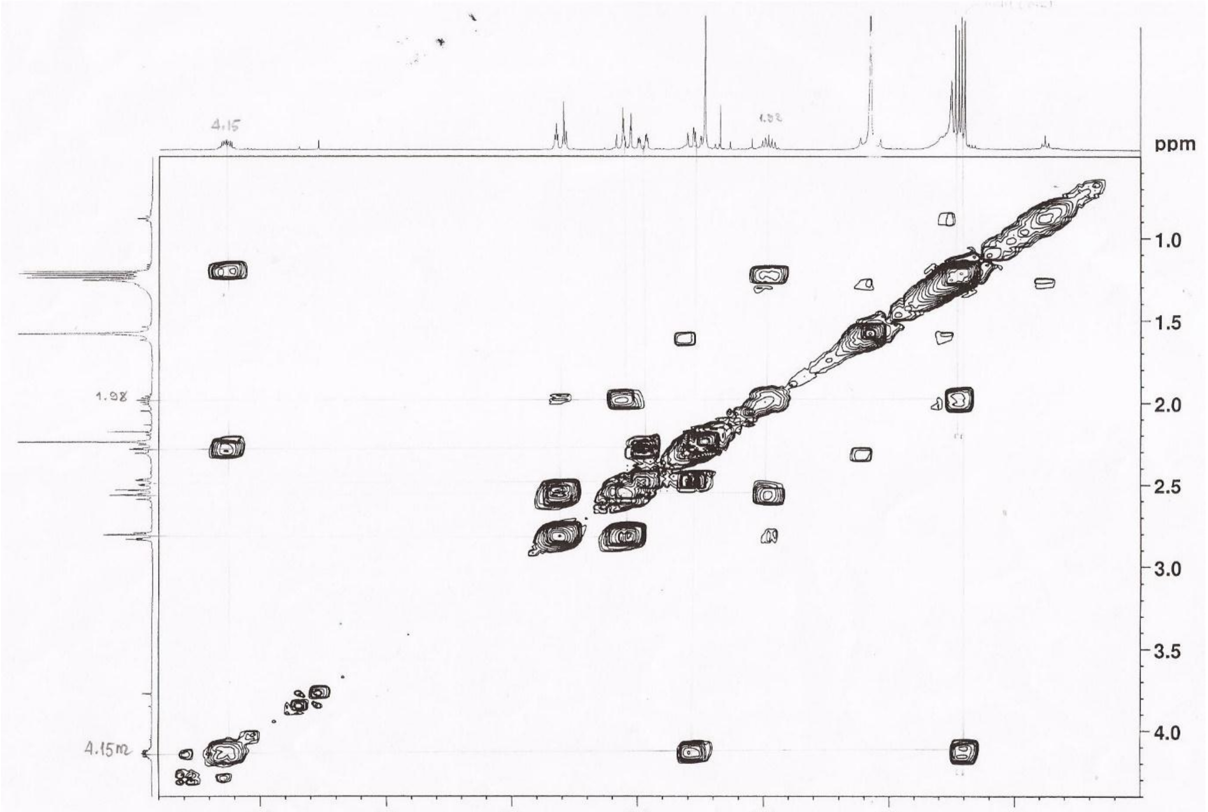

Figure S2. COSY spectrum of compound **1** ($CDCl_3$, 500.13 MHz).

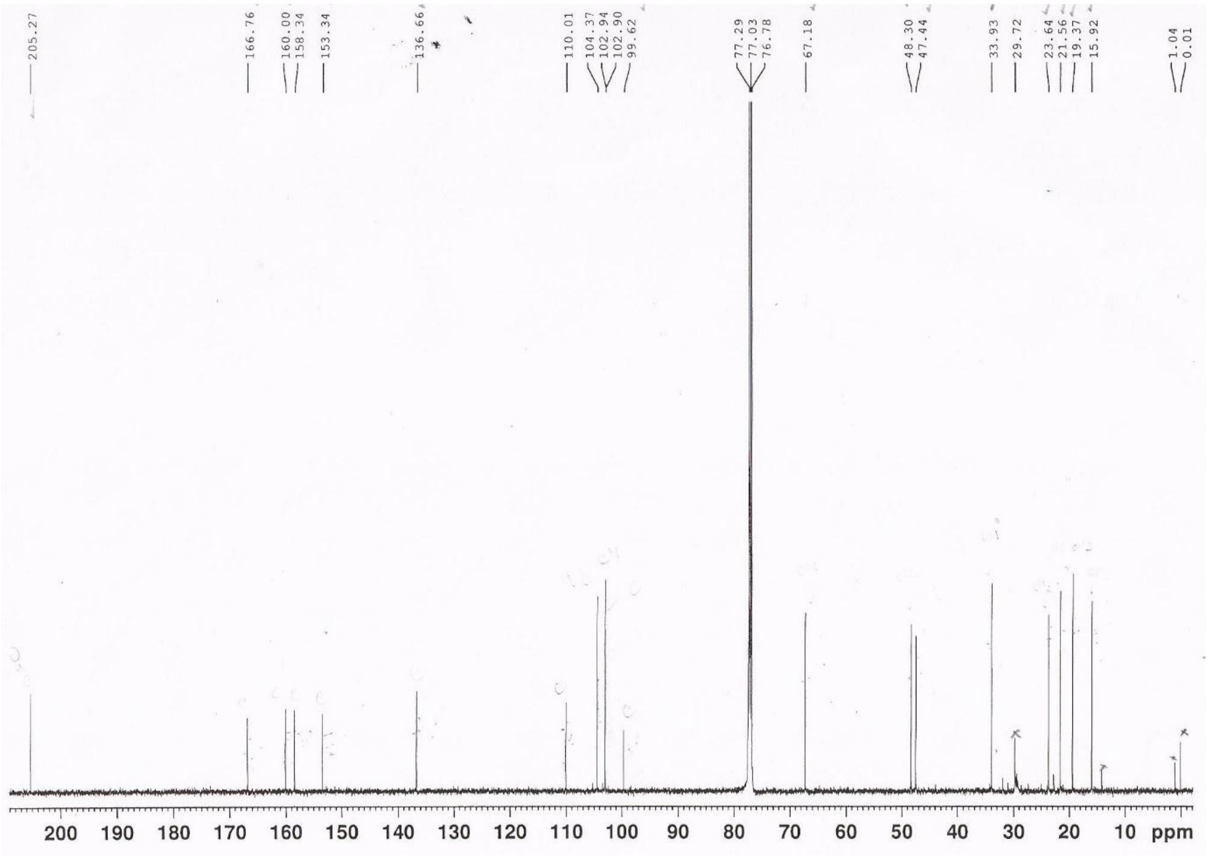

Figure S3. ^{13}C NMR spectrum of compound **1** ($CDCl_3$, 125.8 MHz).

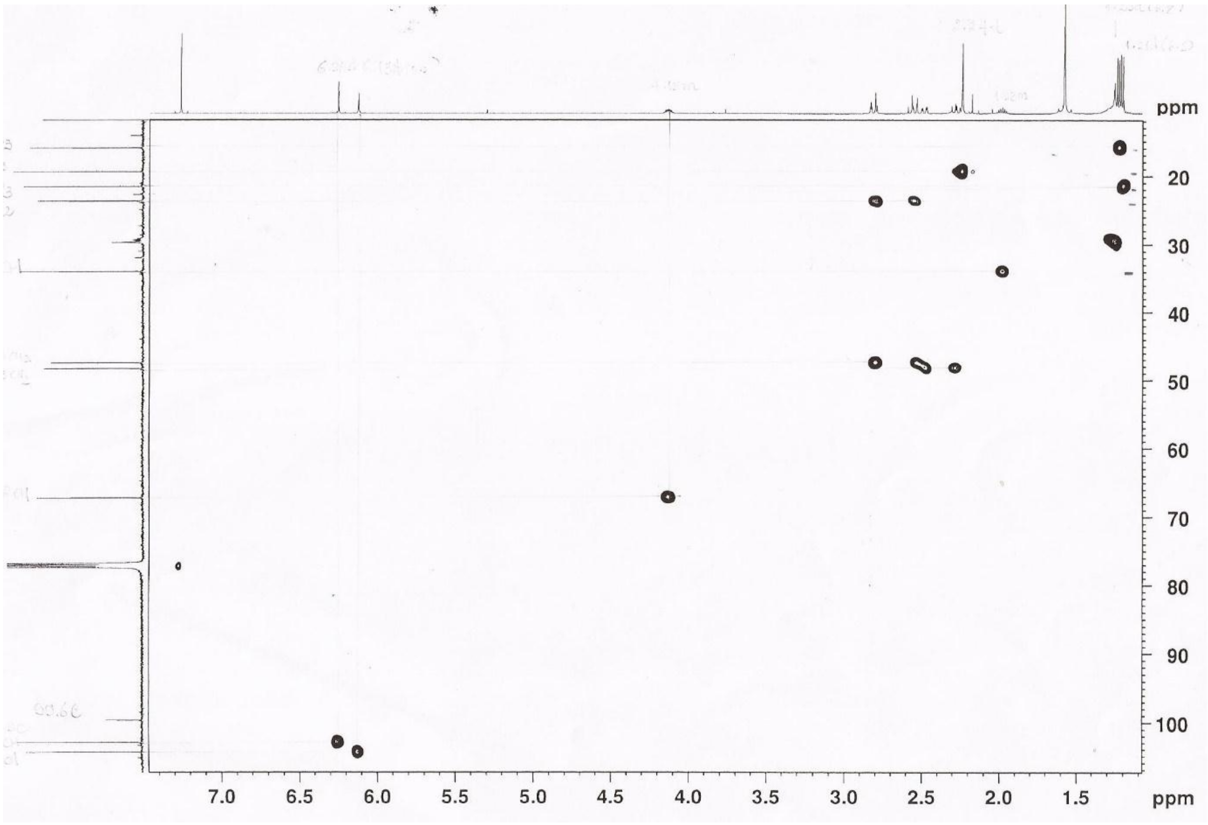

Figure S4. HSQC spectrum of compound **1** ($CDCl_3$, 500.13 MHz).

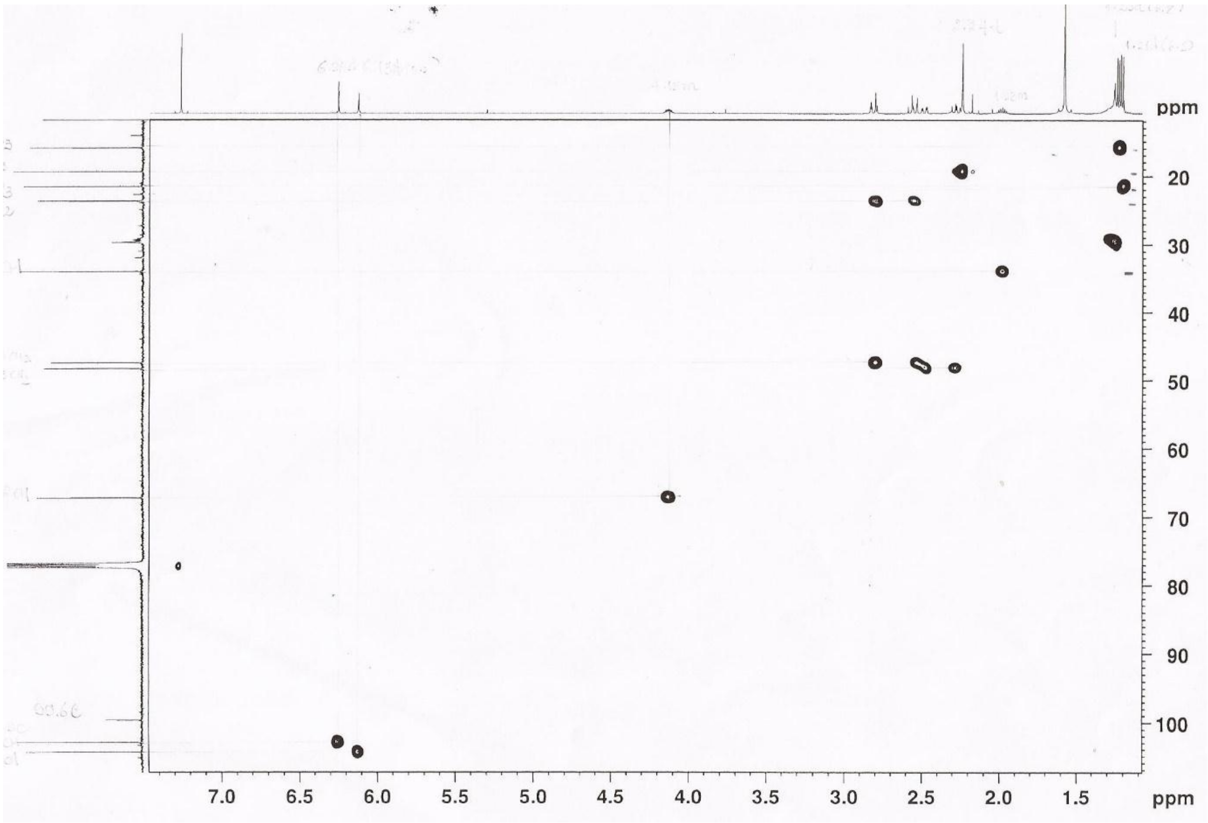

Figure S5. HMBC spectrum of compound **1** ($CDCl_3$, 500.13 MHz).

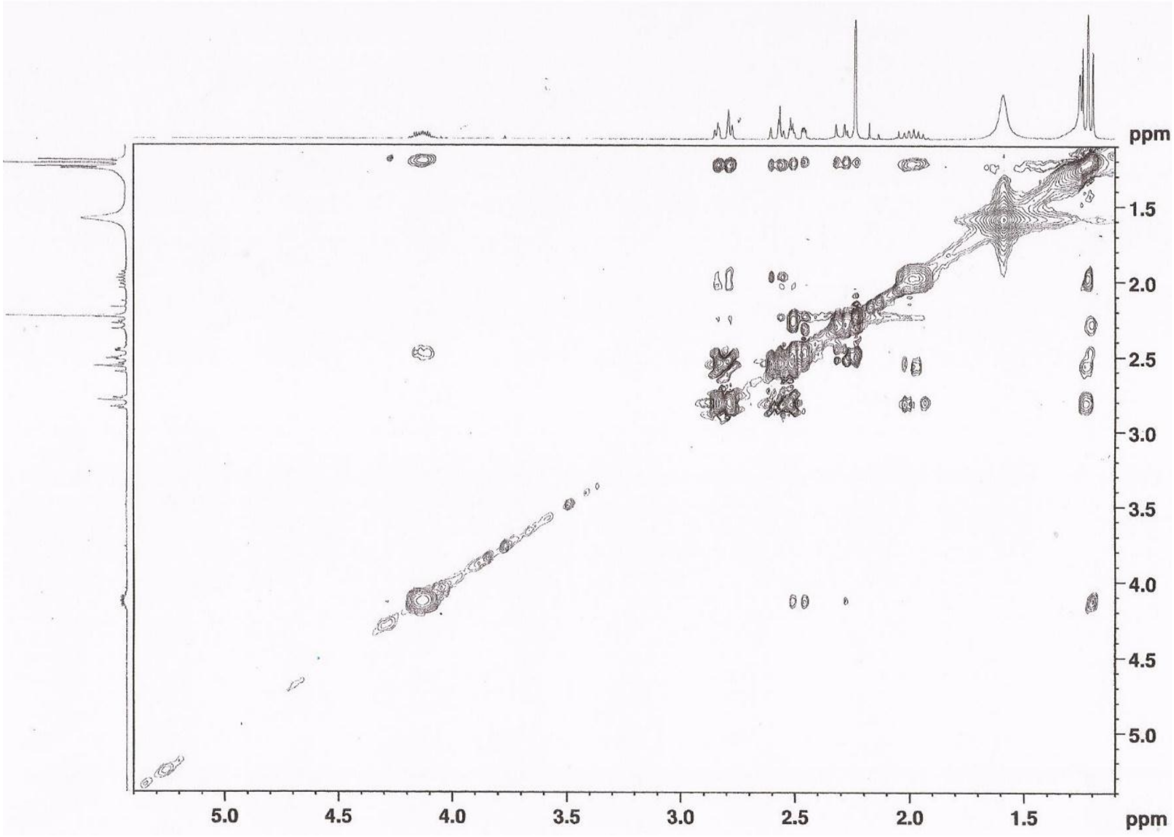

Figure S6. NOESY spectrum of compound **1** ($CDCl_3$, 500.13 MHz).

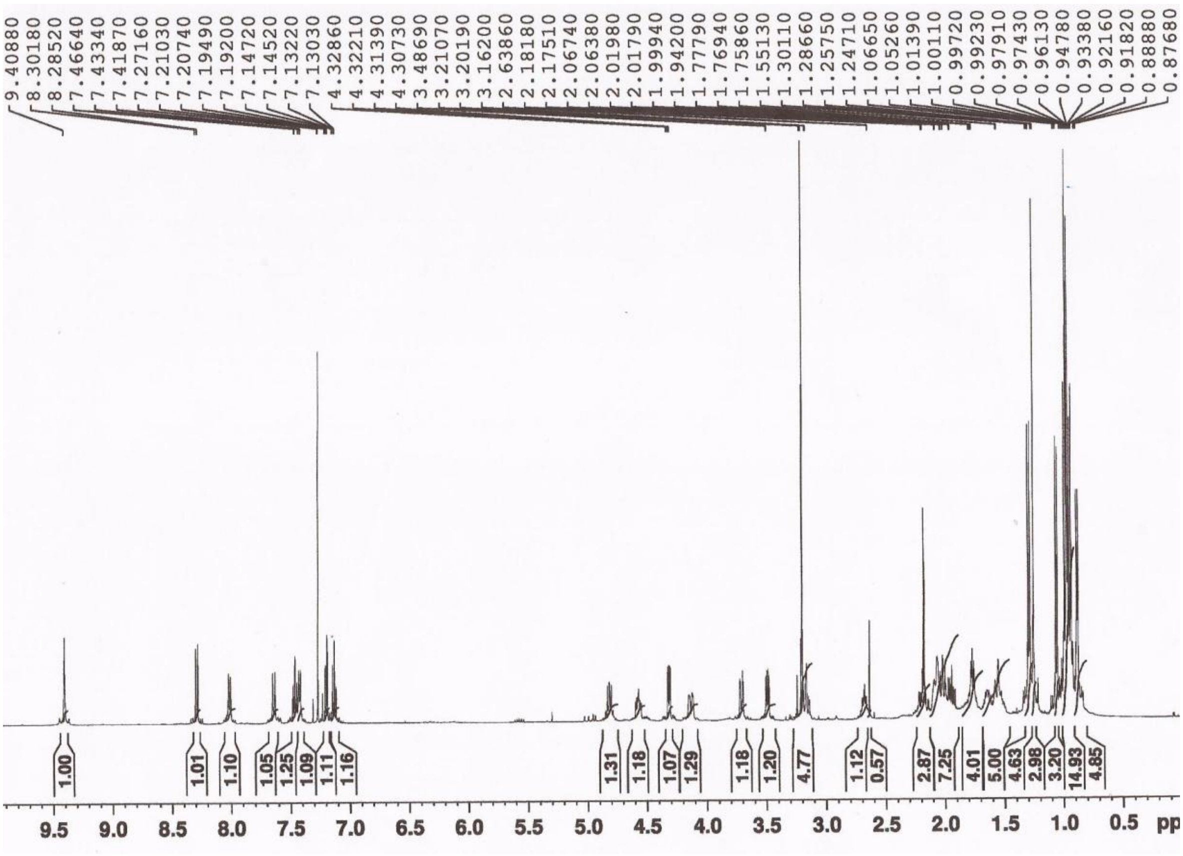

Figure S7. ^{1}H NMR spectrum of compound **2** ($CDCl_3$, 500.13 MHz).

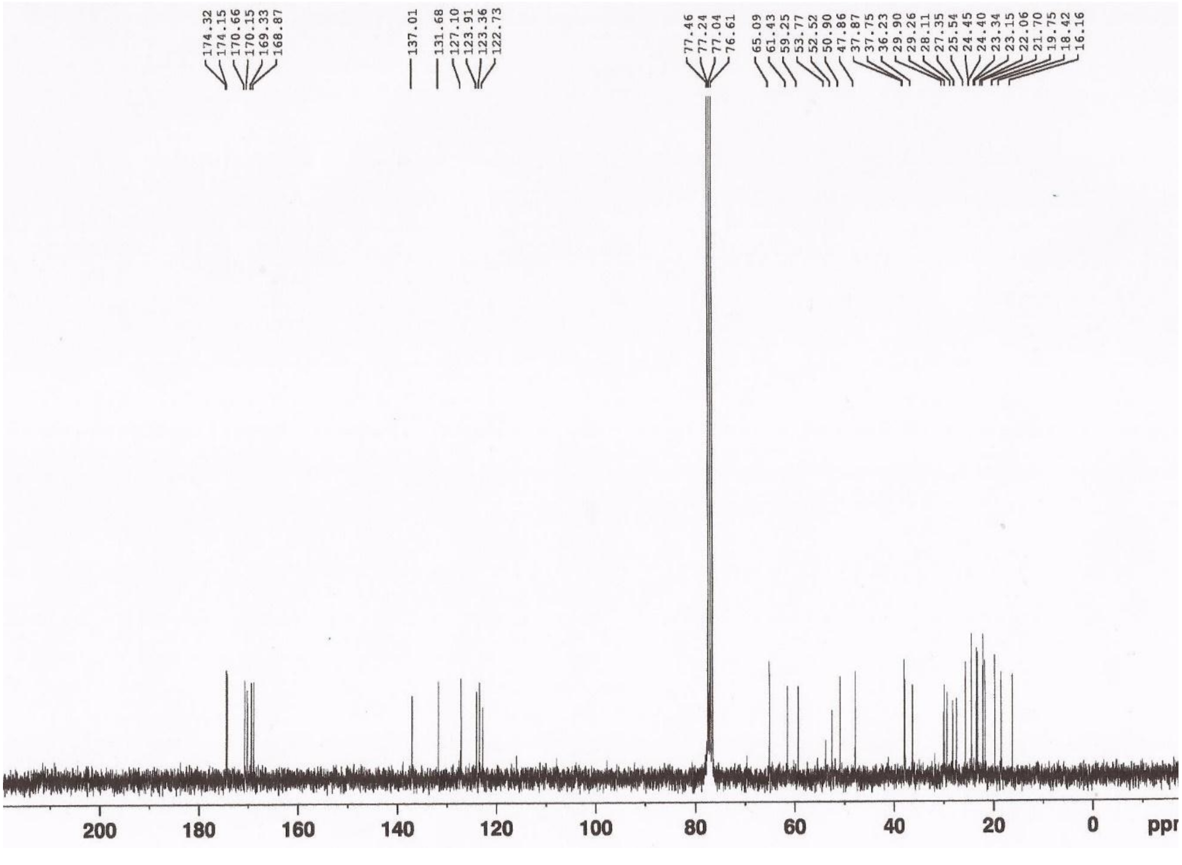

Figure S8. ^{13}C NMR spectrum of compound **2** ($CDCl_3$, 125.8 MHz).

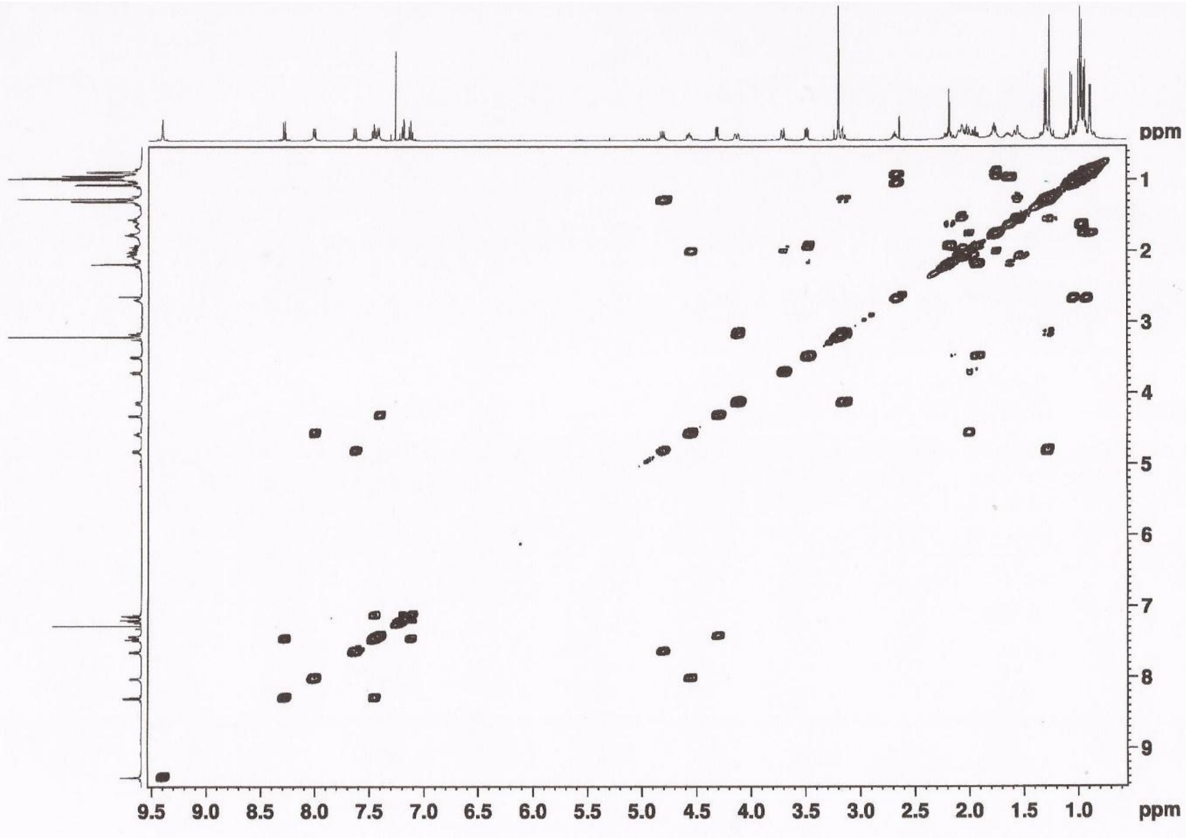

Figure S9. COSY spectrum of compound **2** ($CDCl_3$, 500.13 MHz).

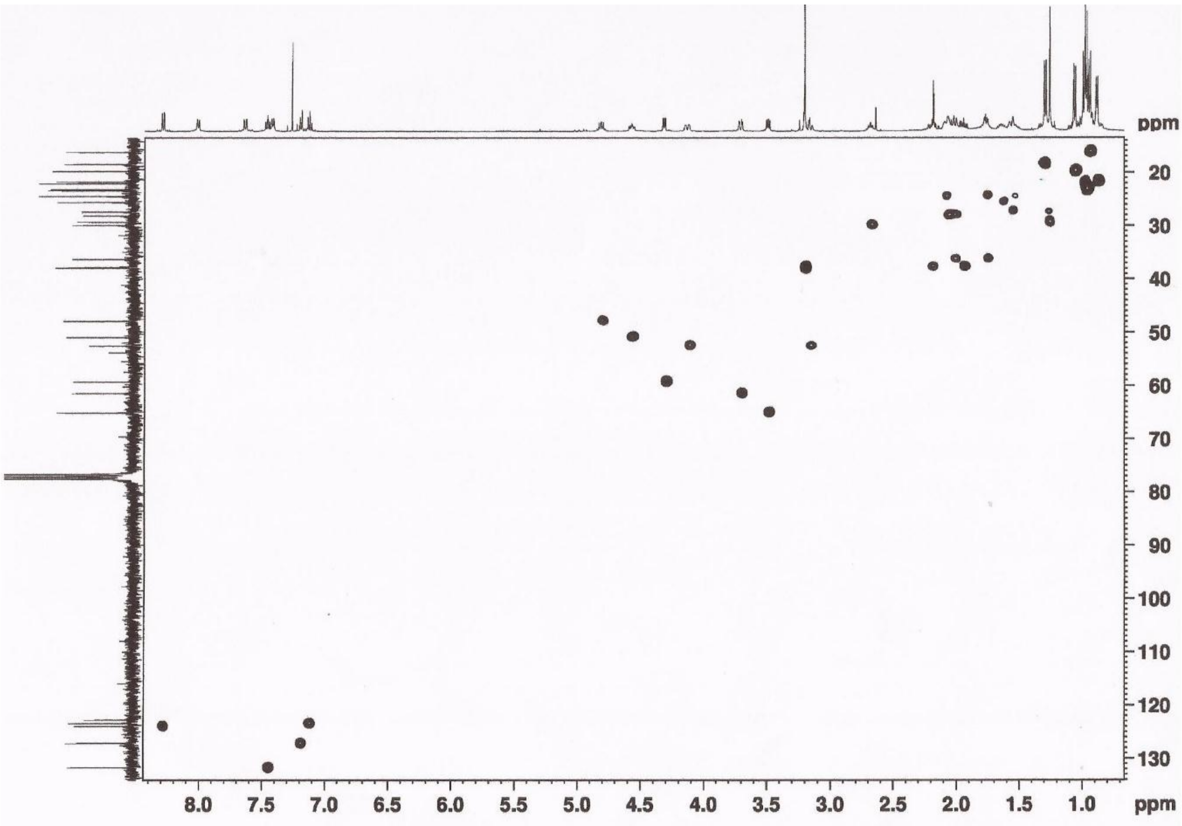

Figure S10. HSQC spectrum of compound **2** ($CDCl_3$, 500.13 MHz).

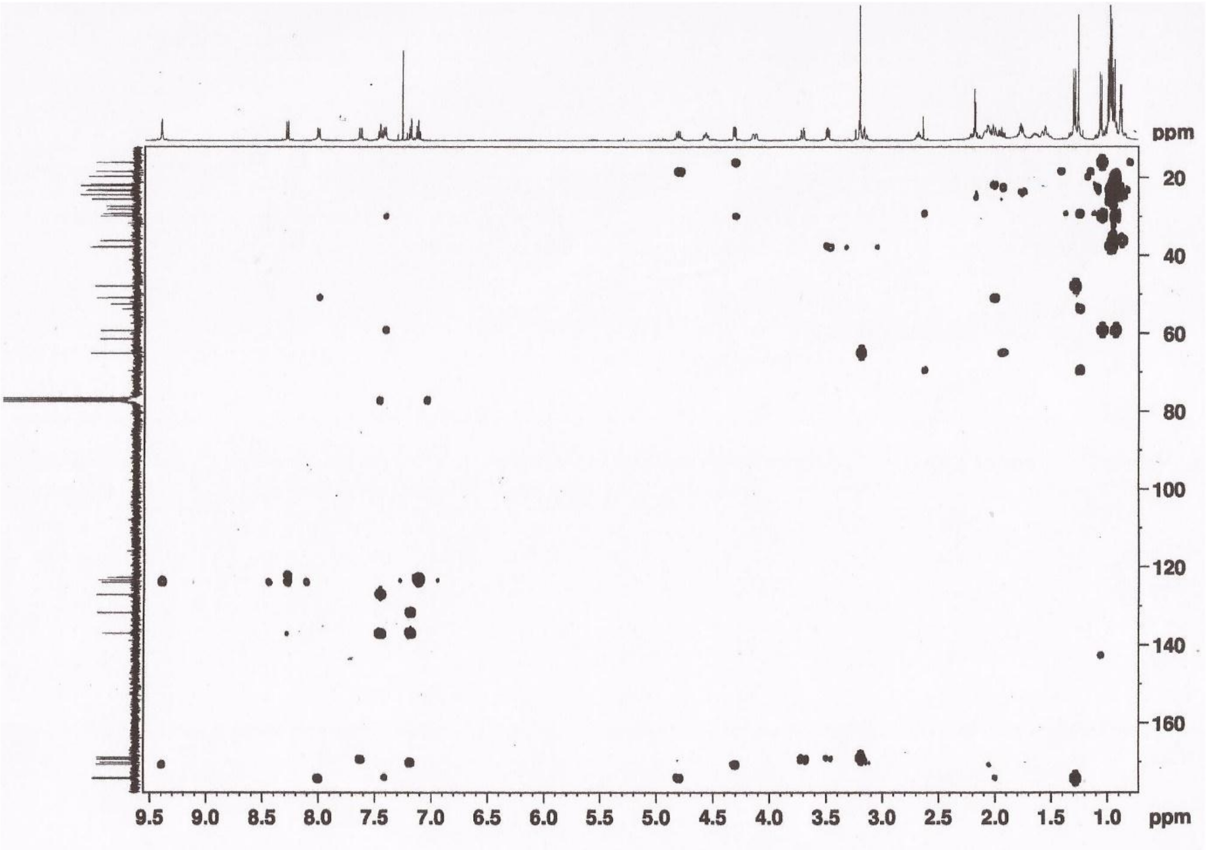

Figure S11. HMBC spectrum of compound **2** ($CDCl_3$, 500.13 MHz).

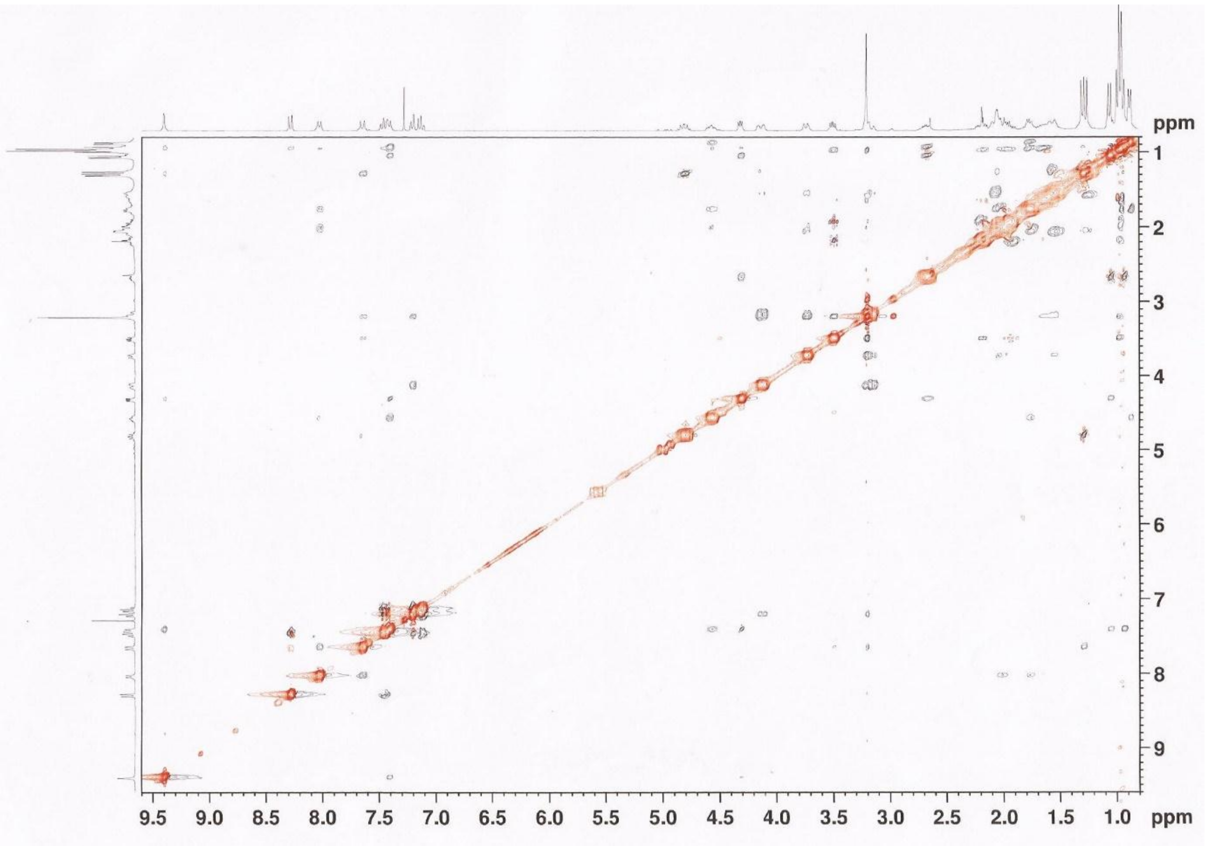

Figure S12. NOESY spectrum of compound **2** ($CDCl_3$, 500.13 MHz).

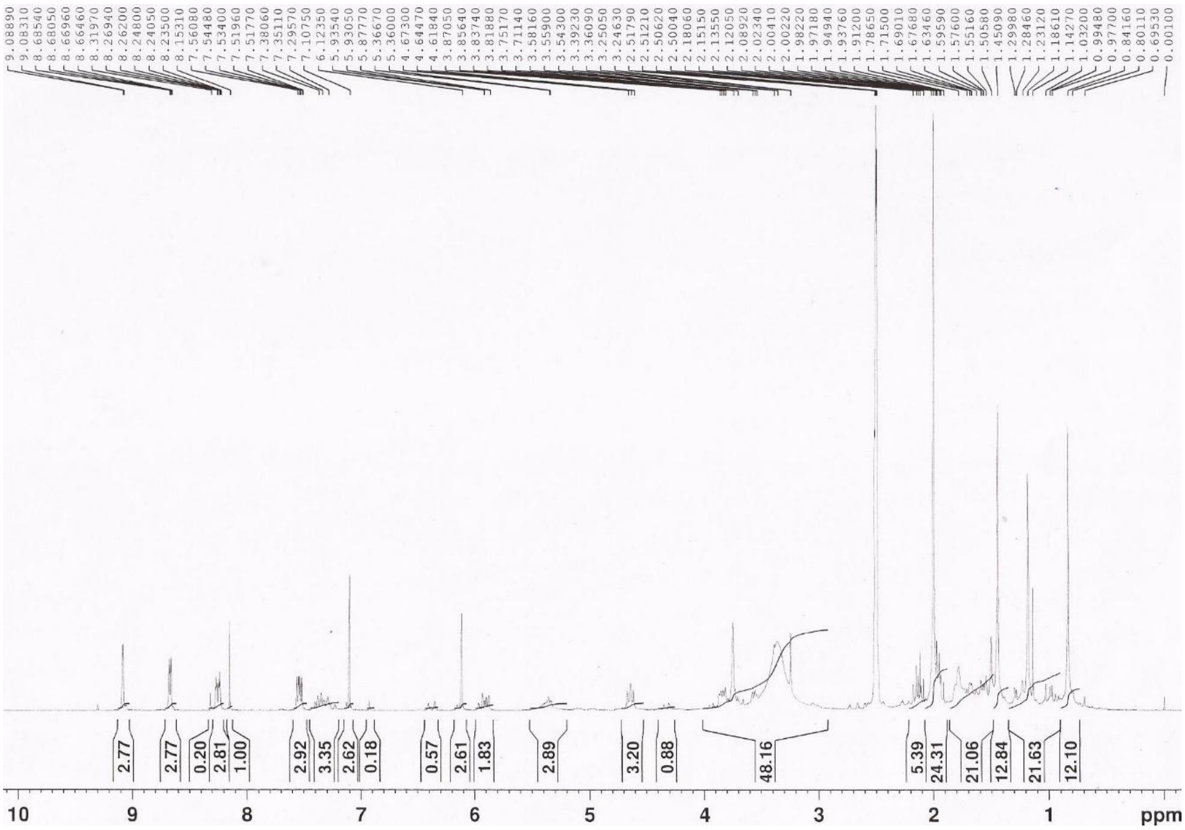

Figure S13. ^{1}H NMR spectrum of compound **3** (DMSO, 300.13 MHz).

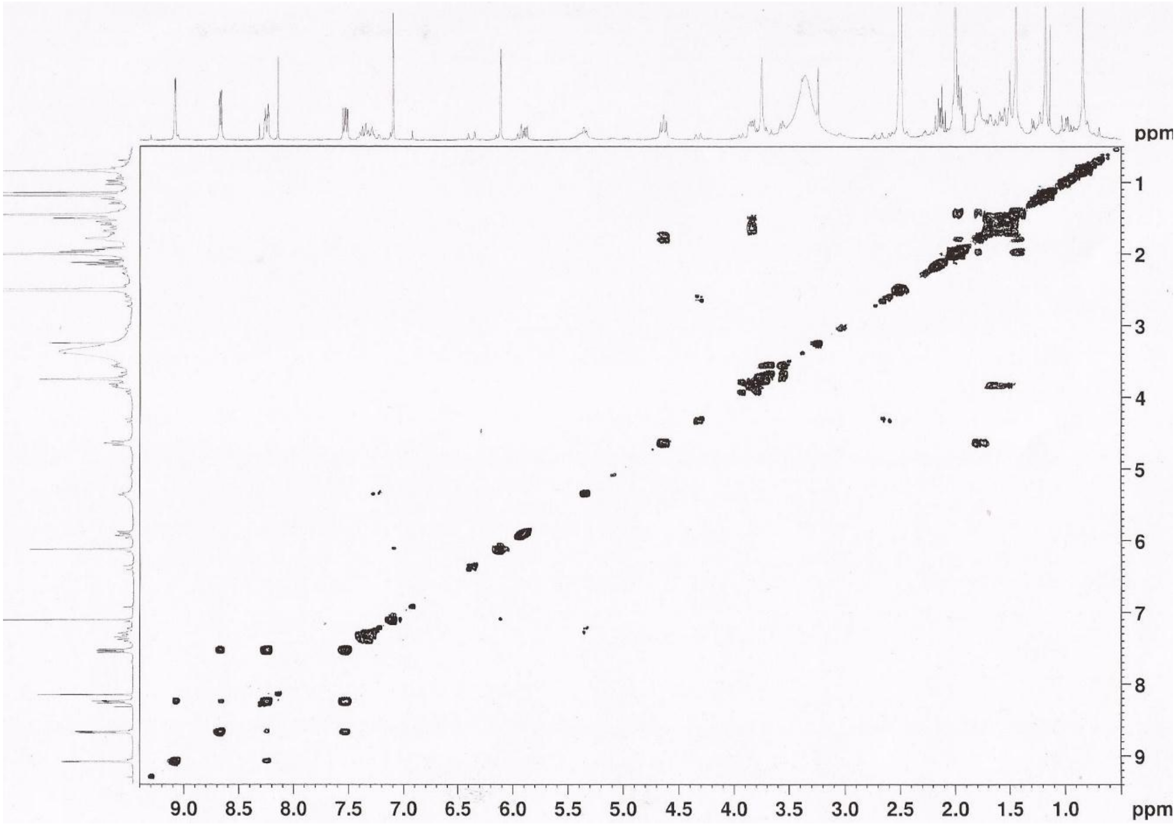

Figure S14. COSY spectrum of compound **3** (DMSO, 300.13 MHz).

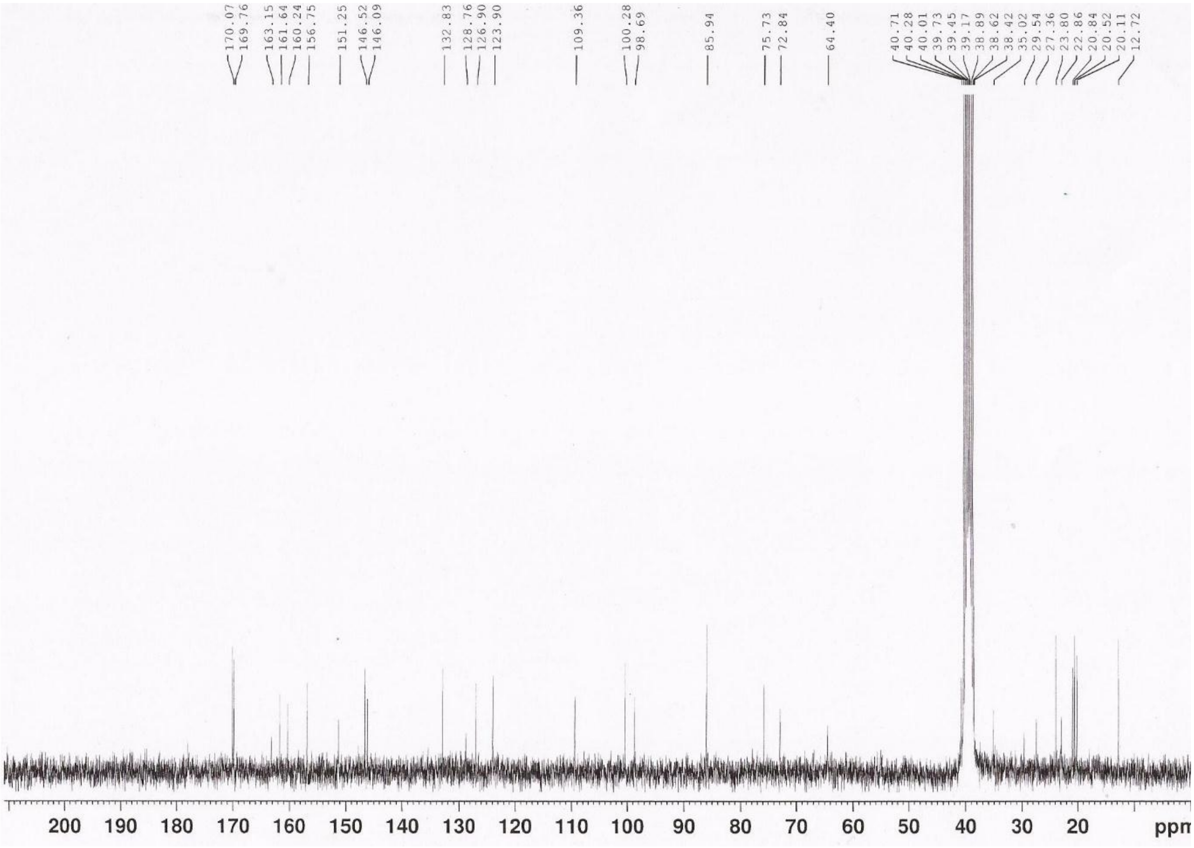

Figure S15. ^{13}C NMR spectrum of compound **3** (DMSO, 75.47 MHz).

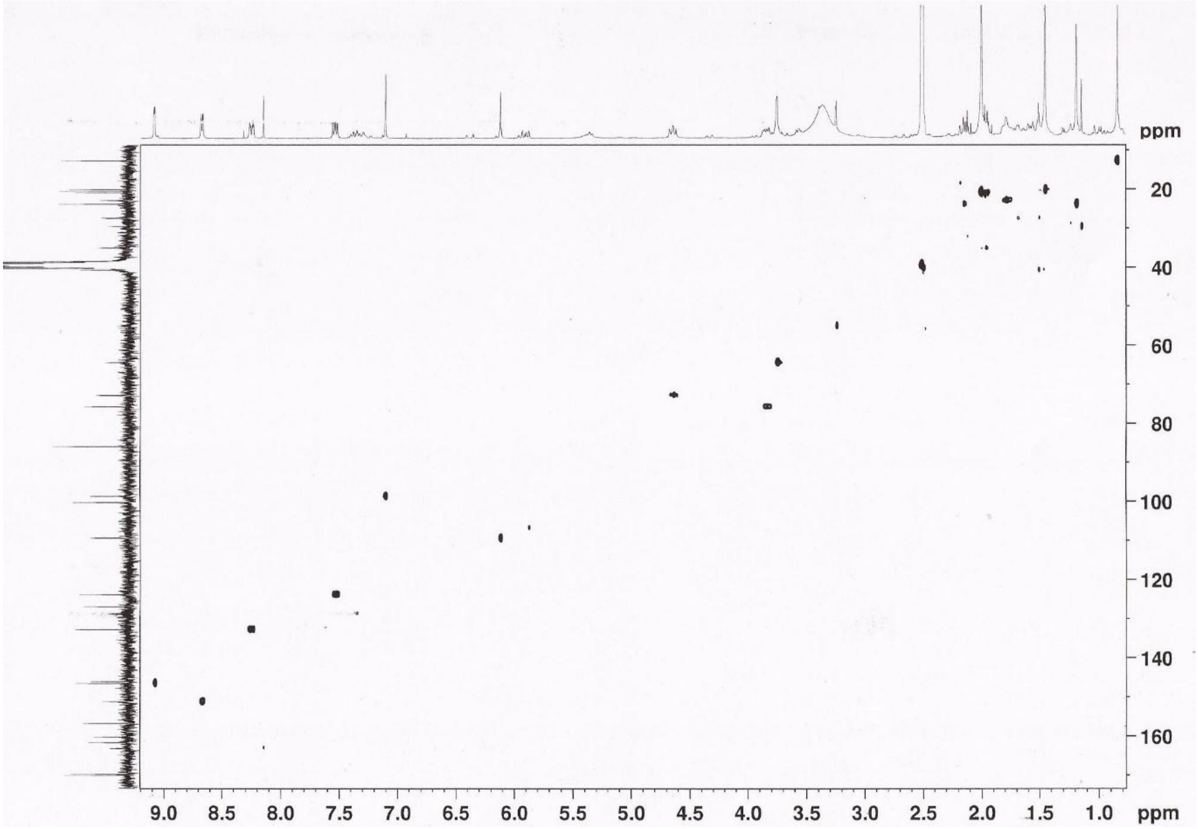

Figure S16. HSQC spectrum of compound **3** (DMSO, 300.13 MHz).

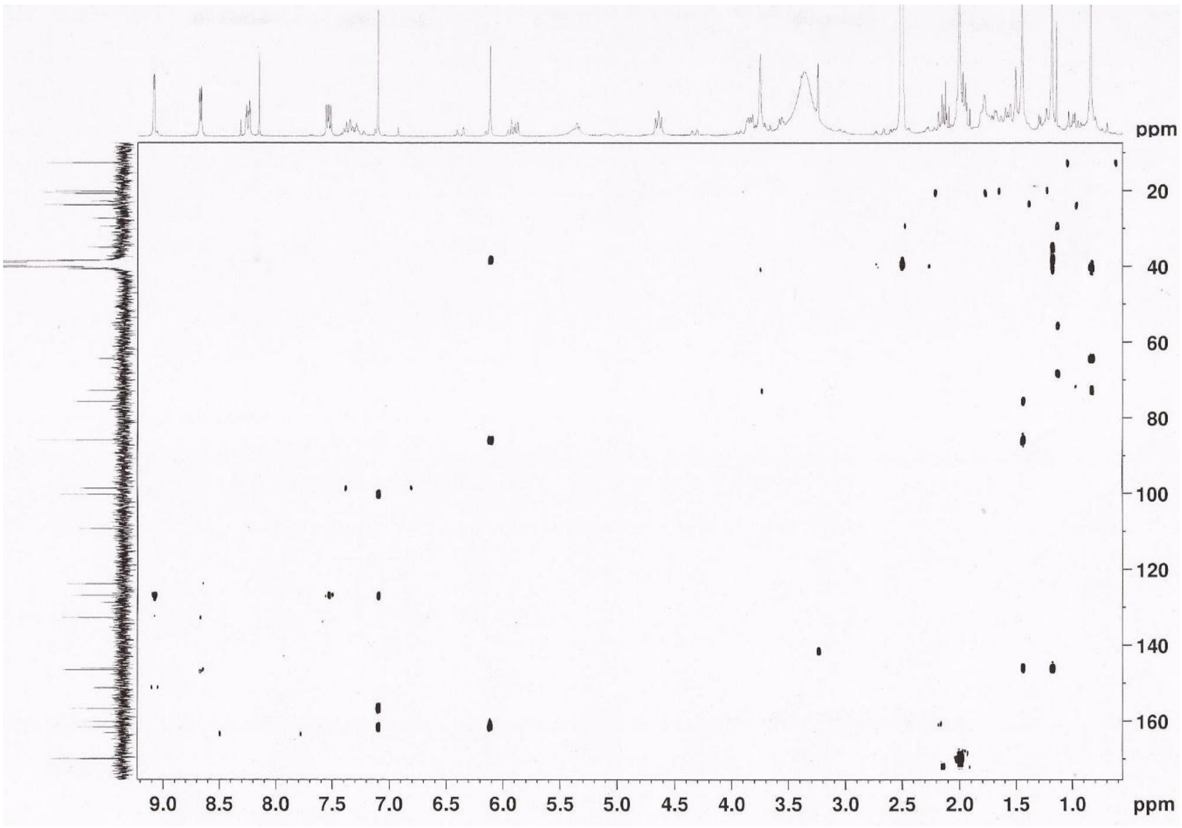

Figure S17. HMBC spectrum of compound **3** (DMSO, 300.13 MHz).

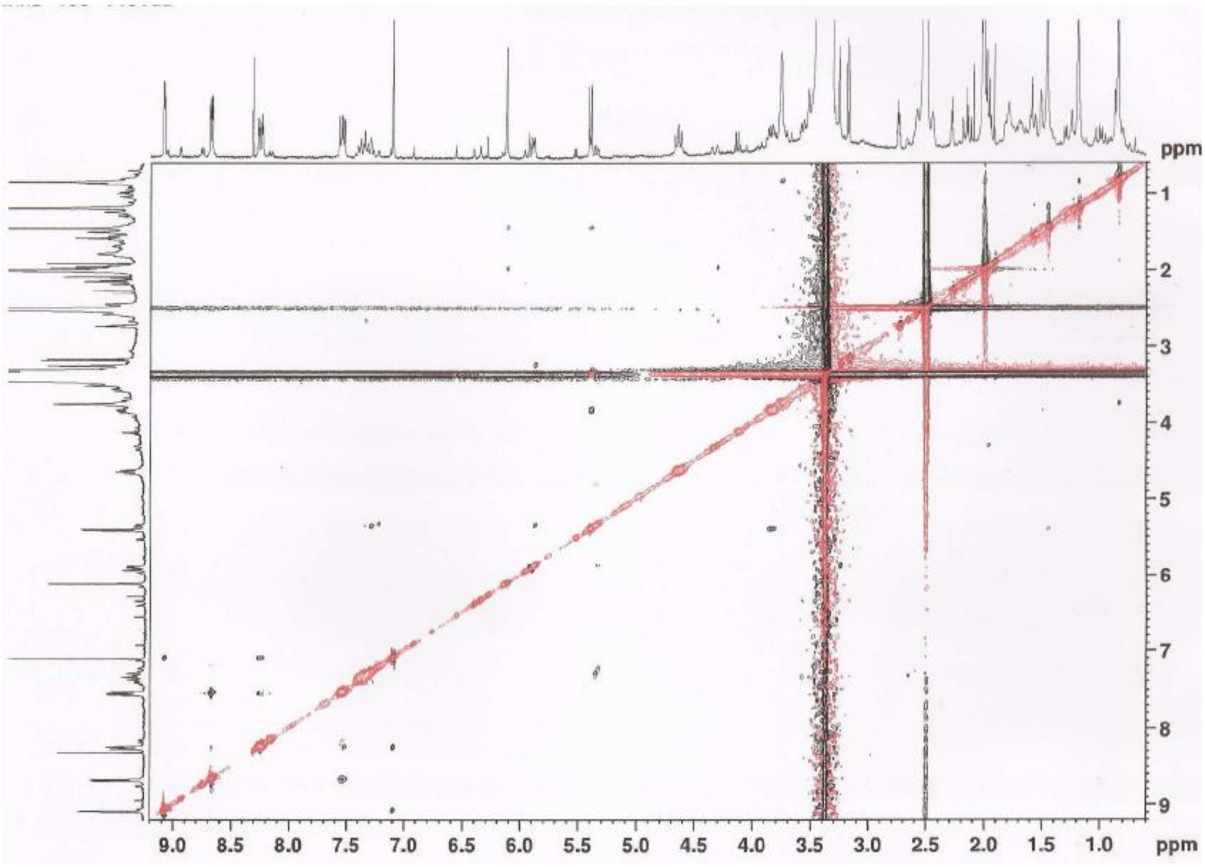

Figure S18. NOESY spectrum of compound **3** (DMSO, 300.13 MHz).

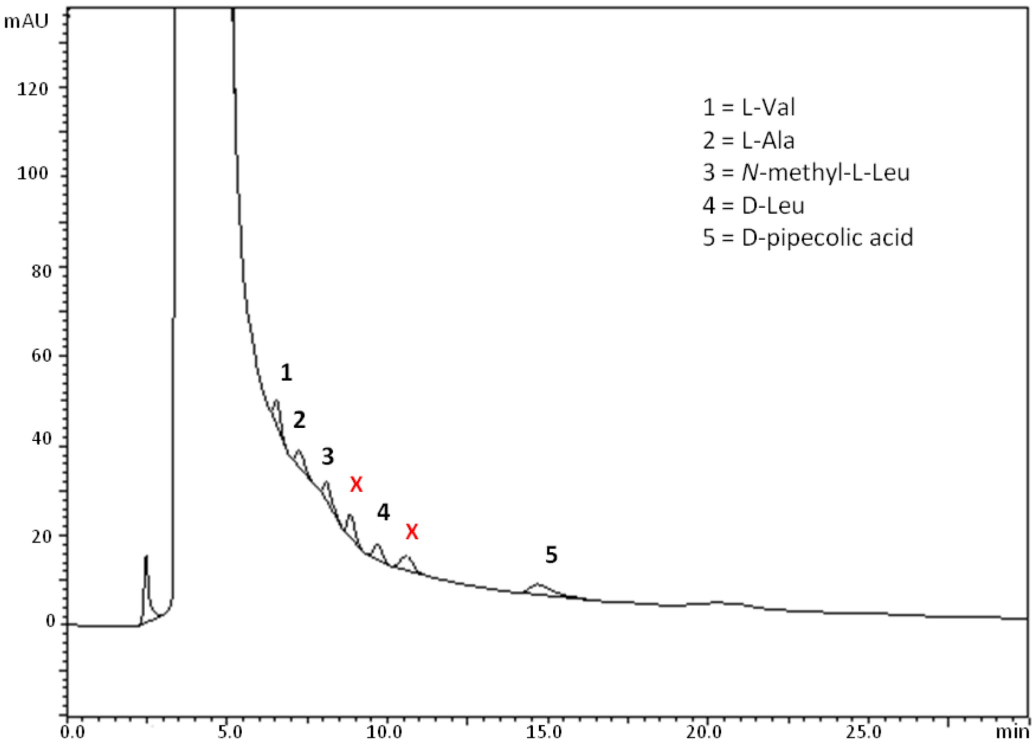

Figure S19. Chromatogram of the acidic hydrolysate of compound **2**. Chromatographic conditions: column, Chirobiotic T; mobile phase, MeOH:H_2O:CH_3CO_2H (70:30:0.02 v/v/v); flow rate, 0.5 mL/min; detection, 210 nm.

Table S1. Chiral HPLC analysis of the acidic hydrolysate of compound **2** *.

	**Retain Time (min)**
L-valine	6.60
D-valine	8.32
L-alanine	7.16
D-alanine	9.36
L-leucine	6.78
D-leucine	9.67
L-pipecolic acid	8.68
D-pipecolic acid	14.67
N-methyl-L-leucine	8.09
Acidic hydrolysate of **2**	6.59, 7.20, 8.09, 8.83, 9.67, 10.57, 14.69
Acidic hydrolysate of **2** + DL-valine (coinjection)	6.61, 7.31, 8.30, 8.10, 8.84, 9.70, 10.50, 14.95
Acidic hydrolysate of **2** + DL-alanine (coinjection)	6.59, 7.19, 8.04, 8.81, 9.37, 9.70, 10.50, 14.90
Acidic hydrolysate of **2** + DL-leucine	6.60, 6.76, 7.26, 8.04, 8.83, 9.67, 10.54, 15.02
Acidic hydrolysate of **2** + DL-pipecolic acid (coinjection)	6.58, 7.20, 8.09, 8.64, 8.84, 9.77, 10.64, 14.64
Acidic hydrolysate of **2** + *N*-methyl-L-leucine (coinjection)	6.59, 7.20, 8.09, 8.83, 9.67, 10.57, 14.69

* Chromatographic conditions: column, Chirobiotic T; mobile phase, methanol:water:acetic acid (70:30:0.02 v/v/v); flow rate, 0.5 mL/min; detection, 210 nm.

© 2015 by the authors; licensee MDPI, Basel, Switzerland. This article is an open access article distributed under the terms and conditions of the Creative Commons Attribution license (http://creativecommons.org/licenses/by/4.0/).
